# Implementing women’s sexual and reproductive health interventions in prisons: a realist review

**DOI:** 10.1136/bmjopen-2025-113940

**Published:** 2026-06-22

**Authors:** Joy Rickard, G J Melendez-Torres

**Affiliations:** 1 University of Exeter, Exeter, UK

**Keywords:** Prisons, Implementation Science, Mass Screening, PUBLIC HEALTH, Preventive Health Services, Sexually Transmitted Disease

## Abstract

**Abstract:**

**Objectives:**

To explore what works for whom, how and why when implementing women’s sexual and reproductive health interventions in prisons to understand the barriers and facilitators to implementation and to generate recommendations for policymakers.

**Design:**

Realist review using the Realist And Meta-narrative Evidence Synthesis: Evolving Standards guidelines.

**Data sources:**

We systematically searched Ovid MEDLINE, Global Health, Embase, the Cumulative Index to Nursing and Allied Health Literature (CINAHL) and the American Psychological Association (APA) PsycINFO databases and hand-searched unpublished literature and reference lists, January–June 2025.

**Eligibility criteria for selecting studies:**

Primary studies of implementing women’s sexual and/or reproductive health interventions, including those addressing sexually transmitted infections, cervical health, breast screening, contraception and women’s health holistically. Study populations included people in prisons that detain women in high-income countries.

**Data extraction and synthesis:**

We extracted and analysed data relating to implementation processes using a grounded theory approach and retroductive inference to articulate cross-case Intervention-Context-Actor-Mechanism-Outcome configurations (ICAMOCs) and refine programme theory. We discussed findings in relation to existing theories from the literature to elicit recommendations for policymakers.

**Results:**

Of 4617 deduplicated records, 26 met the inclusion criteria. Ten ICAMOCs were constructed from cross-case analyses, grouped into three themes: (1) planning (teaming, team leadership, assessing needs and capacity, tailoring and planning), (2) doing (piloting, standardisation and support, trauma-informed engagement and peer advocacy) and (3) sustaining (evaluation-adaptation cycles). The ICAMOCs indicated three overarching mechanisms as being key to effective implementation, namely, perceived utility of the intervention, motivation and empowerment.

**Conclusions:**

For women’s sexual and reproductive health interventions to be effective in prisons, everyone involved in implementation needs to perceive the intervention’s benefit and be both motivated and empowered to take action. We recommend policymakers build a resilient and empowered delivery workforce, invest in research partnerships to increase awareness and understanding and promote trauma-informed approaches to women’s healthcare in prisons.

**STRENGTHS AND LIMITATIONS OF THIS STUDY**

- Realist synthesis is well suited to evaluating the implementation of interventions in complex real-world settings such as prisons.
- The inclusion of studies from unpublished literature may lower publication bias but may also lack rigour.
- Only studies from high-income countries published in English since 2010 were included. Furthermore, the predominance of North American studies (n=19) may limit generalisability to England.
- While pragmatism is advised in realist reviews to balance theoretical saturation with the availability of studies and time, further purposive sampling could have been undertaken.

## Introduction

### Rationale

 It has been shown that people in prison are more likely to have complex needs and poorer health outcomes than the general population and that detention can have a harmful, ageing effect.^1^,^5^ Further evidence suggests this disparity may be greater for people in prisons that detain women (PPDW), who are at higher risk of worse outcomes and premature mortality.^6^,^8^ For example, when compared with people in prisons that detain men, substance misuse was found to be more prevalent, and rates of self-harm, attempted suicide and mental health conditions were higher.^5^,^79^ Further studies found higher prevalence of sexually transmitted infections (STIs), menstrual irregularities and amenorrhoea compared with the community and substantially higher rates of dysplasia and cervical cancer.^910 12^,^16^ There is also evidence that cancers are diagnosed later and have lower overall survival in prison settings than in the community.^2 17 18^

The wider determinants of health and crime are largely comparable, and people detained in prisons for women often encounter multiple disadvantages throughout life.^1 2 5 6^ They are more likely than other people in prison and those in the community to experience poverty, housing insecurity, unemployment, sexual/physical violence, commercial sex work, trafficking and multiple sexual partners.^1 6 9 10 12^ In England, one-third spent time in care, two-thirds survived domestic abuse and ethnic minorities are over-represented.^2 5 6^ It has been suggested that women’s sexual and reproductive health is under-represented in research and policymaking and that existing services can be fragmented and difficult to access for marginalised groups.^19 20^ People are therefore more likely to enter prison with low rates of screening and healthcare engagement, perpetuating a high-risk/low-uptake anticorrelation.^221^,^24^ Such complex intersecting factors can lead to compound health inequalities and unmet needs for PPDW.^5 25^

As sites of ‘dynamic exchange’^26^ (p. 239) with marginalised groups, prisons can provide opportunities for people to engage with public health interventions at least equivalent to those available in the community.^6 10 23 27^ It has been suggested this could reduce health inequalities and reoffending, with downstream benefits for the families, communities and health services people in prison return to on release.^1 2 26 28 29^ England’s Chief Medical Officer^5^ recommends the prioritisation of preventive health interventions in prisons, and there is growing international support for improved women’s sexual and reproductive health provision.^6 30^ However, more research in this field is needed.^6 21 31 32^ In England, only 1% of approved prison-based research projects conducted in 2015–2024 addressed reproductive and/or perinatal health.^5^ Internationally, we found very few systematic and scoping reviews of relevance, and those available describe multiple service gaps and inadequacies, particularly for contraception.^1533^,^38^ A recent National Health Service (NHS) England review found that, despite some examples of good practice, women’s sexual and reproductive health is systemically underserved in prisons.^6^ Improvements in cervical screening uptake, for example, have been shown to plateau considerably below national coverage and targets.^24^

In contrast, there is good evidence that community-based women’s sexual and reproductive health interventions are effective.^39^,^43^ However, the uptake and sustainability of complex interventions are highly context dependent, and implementation strategies used in other settings may not be transferable to prisons.^44 45^ Service reviews characterise England’s prison infrastructure as ‘ageing…designed for a young male population’^5^ (p. 10) and ‘unfit for purpose’^6^ (Section 2.10) for people detained in prisons for women, who constitute <5% of the population.^46^ The evidence suggests that high demand for urgent care can make overburdened, under-resourced health services reactive not proactive and post-pandemic recovery of screening services has been slow.^1 17 47^ Short sentences and geographical displacement may also limit the timeframe for action and present challenges for follow-up.^6 33^ To bridge the evidence-practice gap, there is a need to identify the conditions required for effective and sustainable operationalisation of women’s sexual and reproductive health interventions in the real-world settings of prisons.^44 45 47^

### Rationale for realist synthesis

Complex public health interventions use strategies and resources to improve population health.^48 49^ However, in reality, they interact dynamically with the open social systems in which they are implemented, making outcomes uncertain.^49 50^ That is, multiple contextual factors determine the activation and intensity of multiple mechanisms, which determines the direction and magnitude of multiple outcomes.^51^,^53^ So, an intervention’s impact may differ when implemented for, and by, different actors in a different time and space. This generative model of causality forms the philosophical basis for realist inquiry.^49^

Where traditional systematic reviews focus on internal validity, realist methods are better suited to evaluating external validity by considering interventions within their delivery contexts to better understand facilitators and barriers along the implementation pathway and identify effective strategies to navigate them.^4547^,^49^ This is achieved through ‘retroductive theorising’, working backwards from observed outcomes to infer underlying causal mechanisms.^53^,^55^ In realist synthesis, initial programme theories (IPT) provide high-level hypotheses of how interventions should work.^54 56 57^ Observations from primary studies are then used to form context-mechanism-outcome (CMO) configurations to test and refine the programme theory (PT).^56^,^59^ Middle-range theories can both inform IPT and explain the PT, increasing external validity (see table 1 for key terms).^54 59^ Adopting this approach, the objective of this review was to provide post hoc, theory-based insights for policymakers into what works for whom, how and why when implementing women’s sexual and reproductive health interventions in prisons.^45 47^

**Table 1:** Key concepts in realist reviews

Concept		Definition
Explanatory factors	Intervention (I)	Any resources, strategies or activities used in the design, implementation or evaluation of an intervention.^105^,^107^
	Context (C)	The particular set of circumstances in which the intervention is implemented.^61 106^
	Actor (A)	An individual, group or organisation involved in one or more aspects of the implementation.^61 106^
	Mechanism (M)	An unseen power, force, response, feedback or interaction with the potential to cause an outcome.^51 106^
	Outcome (O)	An intended or unintended consequence of the intervention.^61^
Theory	Initial programme theory (IPT)	Macro-level; a theory about how the intervention should work.^56 57 108^
	Programme theory (PT)	Micro-level; theories about how and why interventions work; the conditions needed (C) to trigger mechanisms (M) to produce outcomes (O).^49 56 59^
	Middle-range theory (MRT)	Macro-level; abstracted theories that explain the PT.^54 59^

## Materials and methods

This review is reported according to Realist And Meta-narrative Evidence Synthesis: Evolving Standards (RAMESES),^58^ guided by Pawson *et al*’s^49^ five steps, with adaptations drawn from the realist literature.

Informal scoping of the literature by the lead author (JR), and discussion with the second author (GJMT), informed the IPT, the scope of the review and the search strategy.^60^ Next, JR conducted in-depth searches for primary studies and extracted data into Intervention-Context-Actor-Mechanism-Outcome configurations (ICAMOCs)—here, intervention (I) and actor (A) were added as explanatory factors to the CMO heuristic to differentiate intervention from context and specify the individuals/groups/institutions involved (see table 1).^5961^,^63^ Finally, JR discusses the PT to provide recommendations for policymakers.

### Scoping the literature

JR conducted informal, exploratory searches of the literature to (a) identify relevant national and international policies and guidance to inform both the women’s sexual and reproductive health domains for inclusion and the IPT development and (b) scope the availability and nomenclature of primary studies to inform the search strategy.

#### Women’s sexual and reproductive health domains

Women’s health hubs (WHH) have been championed as a model for improving access to women’s health services in the community, including those on probation or community supervision.^5 19^ Therefore, we used the core services defined in the WHH core specification^64^ to identify women’s sexual and reproductive health domains for inclusion. We cross-referenced this with key policy and guidance documents to ensure relevance to England’s prison context.^23 28 30 65 66^ Pregnancy testing received multiple recommendations, so was added. Pessaries and preconception care were not mentioned, so were removed. Menopause was absent from prison recommendations but identified as an overlooked need in a recent NHS England review,^6^ so was retained. The final women’s sexual and reproductive health domains in scope for this review are shown in table 2.

**Table 2:** Women’s sexual and reproductive health domains (those excluded are shown in italics)

Women’s sexual and reproductive health domain	WHH^64^	NHS^23^	WHO^28^	NICE^65^	BASHH^66^	PRI^30^
STI/HIV (screening)	Yes	Yes	Yes	Yes	Yes	Yes
Cervical (screening)	Yes	Yes	Yes	Yes		Yes
Breast (screening)	Yes	Yes	Yes	Yes		Yes
Menstrual problems	Yes		Yes		Yes	Yes
Contraception	Yes		Yes		Yes	
Pregnancy (testing)			Yes	Yes	Yes	
Menopause	Yes					
Preconception care	*Yes*					
Pessary care	*Yes*					

BASHH, British Association of Sexual Health and HIV; NHS, National Health Service; NICE, National Institute for Health and Care Excellence; PRI, Penal Reform International; STI, sexually transmitted infection; WHH, women’s health hubs.

#### Initial programme theory

The Consolidated Framework for Implementation Research (CFIR) is commonly used by implementation scientists to aid systematic analysis.^67^ We therefore formed a conceptual framework based on the five CFIR contextual domains (outer setting, inner setting, intervention, implementation process and actors), plus constructs from the CFIR implementation process domain (teaming, assessing needs/capacity, planning, tailoring, implementing, engaging, evaluating and adapting), modifying terms where appropriate to reflect realist terminology (table 1) and the sensitivity of the review topic. We supplemented this with Proctor *et al*’s^68^ implementation outcomes (appropriateness, acceptability, feasibility, adoption, integration/reach, fidelity, cost-effectiveness and sustainability) and contextual factors of relevance from the prison healthcare guidance documents identified in the initial scoping.^6 23 28 30 65 66^ This conceptual framework forms an IPT of how the implementation of women’s sexual and reproductive health interventions in prisons should work. We include a pictorial representation in online supplemental file 1.

### Searching process

We developed and implemented the search strategy iteratively in January–June 2025, following realist principles.^49^ This included published and unpublished literature and used online searching and ‘snowballing’ citation searching methods. JR performed systematic searches of peer-reviewed journals using Ovid MEDLINE, Global Health, Embase, the Cumulative Index to Nursing and Allied Health Literature (CINAHL) and the American Psychological Association (APA) PsycINFO databases to provide an appropriate spread of the indexed health research literature, including medical, public health and mental health research. Synonymous search terms identified through exploratory searches were aggregated, including multiple alternatives for prisons and people in prison (jail, detention, incarceration, remand, felon, etc) to reflect international and colloquial variations. Terms were combined with Boolean commands (AND/OR/NOT) and wildcard characters, using medical subject heading (MeSH) terms as appropriate (see online supplemental file 1 for full details). We limited searches to 2010 or later, as the United Nations adopted the ‘Bangkok Rules’^30^ for women’s prison healthcare in 2010. JR used EndNote to manage and deduplicate search results and Mendeley Cite to manage citations.

### Selection and appraisal of documents

Informal searches highlighted a scarcity of England-based intervention studies, so all high-income countries were included.^69^ While significant heterogeneity was expected, limiting studies to high-income countries may increase generalisability to England’s prisons.

JR deduplicated results and screened titles and abstracts against inclusion/exclusion criteria (see online supplemental file 1). In brief, interventions implemented in high-income country prisons addressing at least one women’s sexual and reproductive health domain (table 2) and delivered during detention were included. Adult women, people who identify as female and people of diverse gender assigned to be detained in a prison for women, were included (referred to throughout as PPDW). Studies that did not describe or evaluate implementation components, factors or outcomes or were unavailable in English were excluded. JR then conducted full-text screening, another reviewer (see Contributors) rescreened a 10% random sample and discrepancies in decisions were discussed and resolved.

Aligning with realist methods,^49^ JR created a matrix (see table 3) to appraise records for relevance, richness and rigour, using a high-medium-low scale based on concepts described by Dada *et al.*^70^

**Table 3:** Appraisal criteria, based on concepts described by Dada *et al*^70^

Appraisal	High	Medium	Low
Relevance	Direct focus and/or good specificity	Single issue and/or some specificity	Indirect issue and/or little specificity
Richness	Rich description and/or explicit theory	Adequate description and/or little explicit theory	Weak description and/or no explicit theory
Rigour	Credible source and/or transparent	Potential bias and/or somewhat transparent	Clear bias and/or poor transparency

### Data extraction

JR created a Microsoft Excel matrix to capture summary information about included studies, comprising title, author, publication year, country, study type, gender, women’s sexual and reproductive health domain(s), brief description and appraisal rating. A second matrix captured Intervention-Context-Actor-Mechanism-Outcome configurations (ICAMOCs). Data extraction and coding were managed using Delve Tool software.^71^

Data relating to implementation processes were extracted, but those relating to study processes were not. For example, eligibility identification for interventions was, but selection processes for trial participants were not. As England’s prison health services are free-at-point-of-care, factors relating to user fees were not extracted, although any wider implications were considered.

### Analysis and synthesis process

JR analysed studies adopting a grounded theory approach. Potential explanatory factors were identified inductively through open coding, guided by—but not limited to—the IPT.^72^ Any convergence or divergence of outcomes was noted, and interventions reported in multiple studies were cross-referenced. Axial coding was then used to group and refine explanatory factors into case-specific ICAMOCs from which cross-case ICAMOCs were inductively and abductively inferred, and deductively tested, in an iterative process. Thus, working retroductively, causal relationships were articulated and discussed in relation to middle-range theories and the IPT to derive insights.

### Patient and public involvement

No patients or members of the public were involved in the design, conduct, reporting or dissemination of this research.

## Results

### Document flow diagram

**Figure 1:**
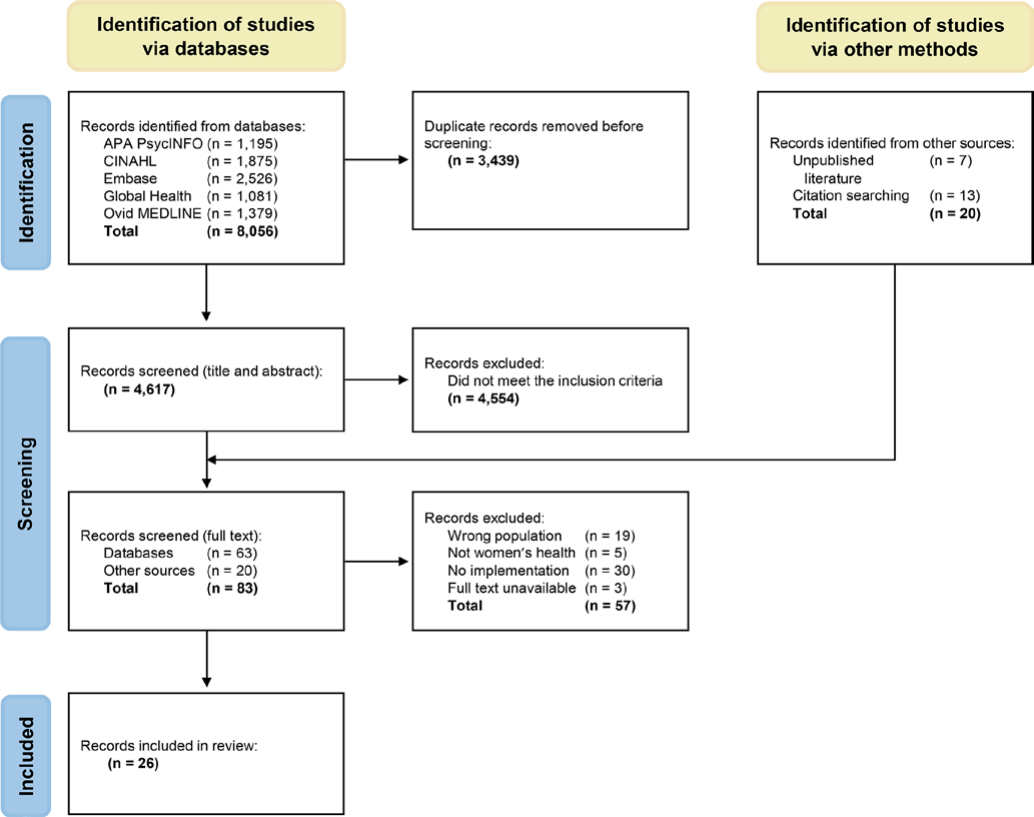
Flow diagram of search results and the selection of records.

Systematic searches identified 4637 deduplicated records, of which 4554 did not meet inclusion criteria. After full-text screening, a further 57 records were excluded, and 26 were included in this review. Figure 1 presents a Preferred Reporting Items for Systematic Reviews and Meta-Analyses (PRISMA) flow diagram of the searching and selection process.

### Study characteristics

Included studies represented five high-income countries: 17 from the USA,^73^,^89^ three from Australia,^90^,^92^ three from England,^93^,^95^ two from Canada^96 97^ and one from Ireland.^98^ There were 12 evaluations,^7374 76 78 79 85 87 90 92^,^95^ seven case studies,^75 77 83 86 88 89 91^ four quantitative,^80^,^8284^ two qualitative^97 98^ and one mixed methods.^96^ Overall, studies were of good quality; none were rated low against all criteria, while two were rated high against all criteria. Table 4 summarises the study characteristics.

**Table 4:** Characteristics of included documents

Author(s), title, year of publication	Study design	Health domain, country, gender	Intervention overview	Appraisal Rel-Ric-Rig
Against Violence and Abuse. An evaluation of Women in Prison’s Health Matters Project. 2019.^93^	Evaluation	Women’s health, England, F	Women’s health advocacy and health promotion programme.	H-H-L
Besney, *et al* Addressing Women’s Unmet Health Care Needs in a Canadian Remand Center: Catalyst for Improved Health? 2018.^96^	Mixed methods	Women’s health, Canada, F	Women’s health programme including testing, education and care.	H-M-H
Cocoros, *et al* Screening for Hepatitis C as a Prevention Enhancement (SHAPE) for HIV: An Integration Pilot Initiative in a Massachusetts County Correctional Facility. 2014.^81^	Quantitative	STI (HCV), USA, M/F	SHAPE HCV screening and education programme (adapted from an existing HIV intervention).	M-M-M
Cole, *et al* Opt-out screening for Chlamydia trachomatis and Neisseria gonorrhoeae in female detainees at Cook County jail in Cole *et al* (2014).^82^	Quantitative	STI, USA, F	Universal, opt-out STI screening programme on reception to prison.	H-M-H
Crowley, *et al* Hepatitis C virus screening and treatment in Irish prisons from nurse managers’ perspectives - a qualitative exploration. 2019.^98^	Qualitative	STI (HCV), Ireland, M/F	Barriers/facilitators to implementing HCV screening programmes.	L-H-H
Dang, *et al* Paired Testing of Sexually Transmitted Infections with Urine Pregnancy Tests in Incarcerated Women. 2021.^80^	Quantitative	STI, pregnancy, USA, F	Opt-out programme pairing STI screening with routine urine pregnancy testing.	H-L-H
Emerson, *et al* Barriers and facilitators of implementing a collaborative HPV vaccine program in an incarcerated population: A case study. 2020.^77^	Case study	STI (HPV), USA, M/F	Opt-in HPV vaccination programme in a prison population.	M-H-M
Fasula, *et al* Project POWER: adapting an evidence-based HIV/STI prevention intervention for incarcerated women. 2013.^79^	Evaluation	STI (inc. HIV), USA, F	Project POWER sexual health education programme (adapted from SAFE).	H-H-M
Harmon, *et al* Routine screening in a California jail: effect of local policy on identification of syphilis in a high-incidence area, 2016–2017. 2020.^83^	Case study	STI (syphilis), USA, F/M	Syphilis screening programme for targeted age groups.	M-M-H
Hesse, *et al* Cancer screening in prisons: lessons for health providers. 2022.^91^	Case study	Breast screening, Australia, M/F	Nurse-led programme to facilitate breast screening uptake in four prisons.	M-L-L
Holton, Hampton. Implementing High-Intensity, Trauma-Informed Sexual Risk Reduction in Women Involved in the Criminal Legal System. 2023.^84^	Quantitative	STI, USA, F	SSE high-intensity trauma-informed educational programme to reduce STI risk.	H-L-H
Johnson, *et al* Feasibility of an HIV/STI Risk-Reduction Program for Incarcerated Women Who Have Experienced Interpersonal Violence. 2014.^85^	Evaluation	STI (inc. HIV), USA, F	Women’s CoOp STI/HIV prevention programme for prisons (adapted from an existing intervention).	H-H-H
Kelly, *et al* Developing a Cancer Prevention Health Education Resource: A Primer of Process and Evaluation. 2020.^76^	Evaluation	Cancer screening, USA, F	Cervical and breast cancer education resource development.	L-H-M
Kennedy, *et al* Case example of a jail-based cancer prevention clinical trial: Social determinants of health framework, novel experimental design, and retention strategies to facilitate long-term follow-up of clinical trial participants. 2023.^75^	Case study	Cervical screening, USA, F	SHE cervical cancer education programme. *Post-detention* e*lements were excluded.*	M-M-H
McNeely, *et al* Expanding Contraceptive Access for Women with Substance Use Disorders: Partnerships McNeely *et al* (2019).^87^	Evaluation	Contraception, USA, F	Contraceptive programme including prison-based education and prison- or community-based LARC fitting.	H-M-M
Mongale, *et al* Development and optimisation of a reception testing protocol designed to eliminate HCV in the UK prison population. 2024.^95^	Evaluation	STI (HCV), England, M/F	HCV screening protocol.	M-M-M
Murphy, *et al* Implementing Preexposure Prophylaxis for HIV Murphy *et al* (2024).^86^	Case study	STI (HIV), USA, M/F	Pre-exposure prophylaxis programme for HIV prevention.	M-M-M
Pankow, *et al* Evaluating Fidelity to a Modified NIATx Process Improvement Pankow *et al* (2018).^74^	Evaluation	STI (HIV), USA, not stated	HIV-STIC protocol for implementing HIV education, testing and care interventions (adapted from NIATx).	H-H-H
Paynter, *et al* The experiences of family planning health professionals providing care to incarcerated patients: A qualitative study. 2025.^97^	Qualitative	Contraception, Canada, F	Barriers/facilitators to contraceptive services for PPDW. *Elements relating to abortion were excluded.*	L-H-H
Phaw, *et al* Prospective evaluation of the impact of repeated whole prison testing for hepatitis C. 2025.^94^	Evaluation	STI (inc. HCV), England, F	High-intensity test and treat for HCV and other STIs in a whole prison population.	M-L-M
Ramaswamy, *et al* The development of a brief jail-based cervical health promotion intervention. 2015.^78^	Evaluation	Cervical screening, USA, F	SHE cervical cancer education programme development.	M-H-H
Sufrin, *et al* Long-Acting Reversible Contraceptives for Incarcerated Women: Feasibility and Safety of On-Site Provision. 2015.^88^	Case study	Contraception, USA, F	Contraceptive programme including counselling and LARC fitting.	M-M-H
Sufrin, *et al* Family planning services for incarcerated women: models for filling an unmet need. 2017.^89^	Case study	Contraception, USA, F	Contraceptive programmes, including family planning, in 4 women’s prisons.	L-M-L
Thornton, *et al* The New Mexico Peer Education Project: Thornton *et al* (2018).^73^	Evaluation	STI (HCV), USA, M/F	NMPEP peer education programme for HCV risk reduction (adapted from Wall Talk).	M-M-M
Wallis, *et al* Hepatitis C virus point-of-care RNA testing: experience from screening an entire high-security Australian prison population over 3 days. 2023.^92^	Evaluation	STI (HCV), Australia, F	High-intensity HCV test and treat programme in a whole prison population.	M-L-L
Winter, *et al* A nurse‐led intervention improved blood‐borne virus testing and vaccination in Winter *et al* (2016).^90^	Evaluation	STI, Australia, M/F	Nurse-led programme to improve the uptake of BBV/STI testing.	M-L-M

BBV, blood-borne viruses; HCV, hepatitis C virus; HIV-STIC, HIV Services and Treatment Implementation in Corrections; LARC, long-acting reversable contraceptive; NIATx, Network for the Improvement of Addiction Treatment; NMPEP, Prisoner Health is Community Health: New Mexico Peer Education Project; Rel-Ric-Rig, relevance, richness, rigour; SAFE, Sexual Awareness for Everyone; SHAPE, Screening for Hepatitis C as a Prevention Enhancement; SHE, Sexual Health Empowerment; SSE, Safer Sex Education; STI, sexually transmitted infection.

Regarding women’s sexual and reproductive health domains, 16 studies addressed STIs, six of which specified hepatitis C virus (HCV), four addressed cervical and/or breast screening (double-counting the Sexual Health Empowerment intervention),^75 78^ four contraception (double-counting a San Francisco jail intervention)^88 89^ and two addressed women’s health holistically. None specified menopause, menstruation or pregnancy testing, although Dang *et al*’s^80^ STI intervention was paired with pregnancy testing. The majority of studies (n=22) evaluated programmatic interventions, and two evaluated protocol interventions. The remaining studies did not evaluate implementation but provided rich insights into relevant contexts and mechanisms, so were included.^97 98^

### Main findings

**Figure 2:**
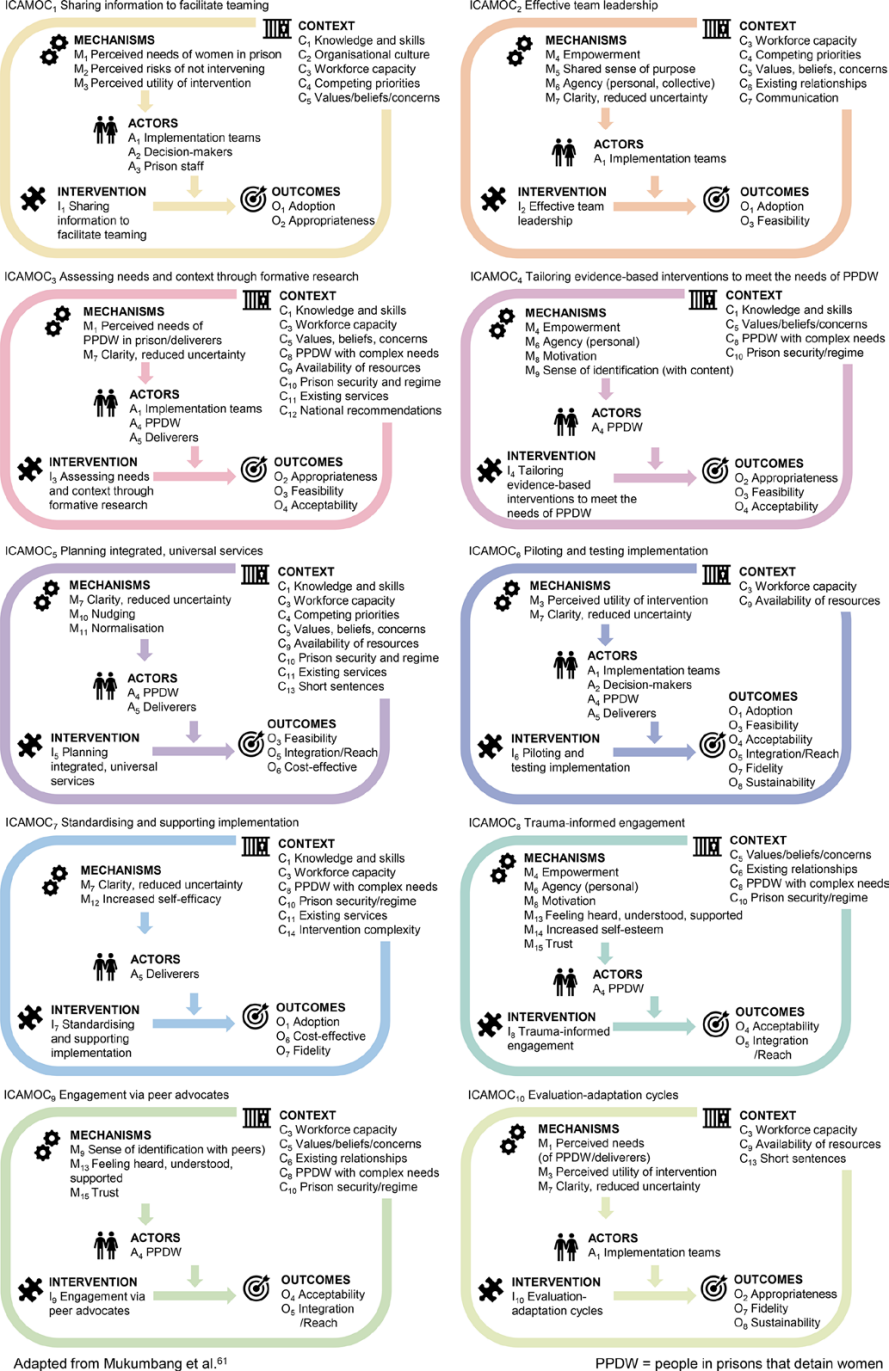
ICAMO configurations 1–10. ICAMO, Intervention-Context-Actor-Mechanism-Outcome; ICAMOC, Intervention-Context-Actor-Mechanism-Outcome configuration; PPDW, people in prisons that detain women.

The analysis elicited ten cross-case ICAMOCs across the three IPT implementation process domains: planning, doing and sustaining. ICAMOCs are described narratively below, pictorially in figure 2, and summarised in table 5 with illustrative extracts from the analysis. Explanatory factors are denoted throughout by unique identifiers in the format (X_y_), for example, the context ‘organisational culture’ is (C_2_).

**Table 5:** Ten ICAMOCs relating to the implementation of women’s sexual and reproductive health interventions in prisons

		Intervention	Context	Actor	Mechanism	Outcome
Planning: teaming	ICAMOC_1_	I_1_ Sharing information to facilitate teaming	C_1_ Knowledge and skills C_2_ Organisational culture C_3_ Workforce capacity C_4_ Competing priorities C_5_ Values, beliefs, concerns	A_1_ Implementation teams A_2_ Decision-makers A_3_ Prison staff	M_1_ Perceived needs (of PPDW) M_2_ Perceived risks of not intervening M_3_ Perceived utility of intervention	O_1_ Adoption (buy-in) O_2_ Appropriateness (perceived suitability, usefulness)
		Illustrative extract: Various administrators and staff initially expressed resistance to providing contraception during incarceration because of the potential suggestion that women were at risk for pregnancy while in custody and because they believed ‘It is not our problem’. Opinion changed when clinicians presented data demonstrating that over half the pregnant women at the facility with prior incarcerations had conceived within 3 months of release (… and)discussed different birth control methods, [and]) post-release challenges for women in accessing family planning services.^89^ (p. 13)				
	ICAMOC_2_	I_2_ Effective team leadership	C_3_ Workforce capacity C_4_ Competing priorities C_5_ Values, beliefs, concerns C_6_ Existing relationships C_7_ Communication	A_1_ Implementation teams	M_4_ Empowerment M_5_ Shared sense of purpose M_6_ Agency (personal, collective) M_7_ Clarity, reduced uncertainty	O_1_ Adoption (buy-in) O_3_ Feasibility
		Illustrative extract: (…)undertaking inter-organisational change with the modified NIATx approach required consistent, meaningful communication between leaders and team members. Change team leadership communication issues were linked to a host of problems, such as scheduling team meetings, delayed change cycles and even a lack of understanding among team members about the HIV-STIC process improvement strategy itself.^74^ (pp. 198–199)				
Planning: assessing	ICAMOC_3_	I_3_ Assessing needs and context through formative research	C_1_ Knowledge and skills C_3_ Workforce capacity C_5_ Values, beliefs, concerns C_8_ PPDW with complex needs C_9_ Availability of resources C_10_ Prison security and regime C_11_ Existing services C_12_ National recommendations	A_1_ Implementation teams A_2_ Decision-makers A_4_ PPDW A_5_ Deliverers	M_1_ Perceived needs (of PPDW /deliverers) M_7_ Clarity, reduced uncertainty	O_2_ Appropriateness (compatibility, relevance) O_3_ Feasibility O_4_ Acceptability (satisfaction with content)
		Illustrative extract: (…)the principal investigator (PI) approached members of the NCDOC and prison administration to assess their capacity and interest in the project(… and)formative research was conducted to gather specific information for adapting Project SAFE for women prisoners.^79^ (pp. 205–206)				
Planning: tailoring, planning	ICAMOC_4_	I_4_ Tailoring evidence-based interventions to meet the needs of PPDW	C_1_ Knowledge and skills C_5_ Values, beliefs, concerns C_8_ PPDW with complex needs C_10_ Prison security and regime	A_4_ PPDW	M_4_ Empowerment M_6_ Agency (personal) M_8_ Motivation M_9_ Sense of identification (with content)	O_2_ Appropriateness (suitability) O_3_ Feasibility O_4_ Acceptability (satisfaction with content, credibility)
		Illustrative extract: Like most educational programmes, we felt that a cervical health promotion intervention for incarcerated women would have to be tailored specifically to the women’s educational, social and cultural backgrounds—balancing delivery of information and empowerment for health behaviour change against the real structural constraints of their criminal justice involvement.^78^ (p. 433)				
	ICAMOC_5_	I_5_ Planning integrated, universal services	C_1_ Knowledge and skills C_3_ Workforce capacity C_4_ Competing priorities C_5_ Values, beliefs, concerns C_9_ Availability of resources C_10_ Prison security and regime C_11_ Existing services C_13_ Short sentences	A_4_ PPDW A_5_ Deliverers	M_7_ Clarity, reduced uncertainty M_10_ Nudging M_11_ Normalisation	O_3_ Feasibility O_5_ Integration and reach O_6_ Cost-effectiveness
		Illustrative extract: (…)paired screening of GC/CT with routine urine pregnancy test leads to an increased number of completed tests and a higher detection of GC and CT infections that would have otherwise been missed. Compared with testing rates before paired testing where GC/CT tests were ordered based on clinical suspicion, implementation of paired testing increased monthly testing rates by 4.7-fold.^80^ (pp. S21–22)				
Doing: implementing	ICAMOC_6_	I_6_ Piloting and testing implementation	C_3_ Workforce capacity C_9_ Availability of resources	A_1_ Implementation teams A_2_ Decision-makers A_4_ PPDW A_5_ Deliverers	M_3_ Perceived utility of intervention M_7_ Clarity, reduced uncertainty	O_1_ Adoption (intention to implement) O_3_ Feasibility O_4_ Acceptability (of content) O_5_ Integration and reach (integration) O_7_ Fidelity (delivery as intended) O_8_ Sustainability
		Illustrative extract: As the trial went on, we learnt ways to facilitate condom skills practice in the prisons that increased participants’ level of comfort, including using humour, having the interventionists model the skills in group before having women try them, and adjusting the timing of the condom skills practice so the practices did not occur after discussing violence or while practising grounding skills, as this was triggering for some women. By the end of the trial, the condom skills practice in the prison facilities was smooth, feasible, acceptable and perceived as helpful by participants.^85^ (p. 3259)				
	ICAMOC_7_	I_7_ Standardising and supporting implementation	C_1_ Knowledge and skills C_3_ Workforce capacity C_8_ PPDW with complex needs C_10_ Prison security and regime C_11_ Existing services C_14_ Intervention complexity	A_5_ Deliverers	M_7_ Clarity, reduced uncertainty M_12_ Increased self-efficacy	O_1_ Adoption (intention to implement) O_6_ Cost-effectiveness O_7_ Fidelity (delivery as intended)
		Illustrative extract: (…)no additional human resources were required; instead, the current staff who were already trained in STI screening were trained in a new protocol and script.^82^ (p. 162)				
Doing: engaging	ICAMOC_8_	I_8_ Trauma-informed engagement	C_5_ Values, beliefs, concerns C_6_ Existing relationships C_8_ PPDW with complex needs C_10_ Prison security and regime	A_4_ PPDW	M_4_ Empowerment M_6_ Agency (personal) M_8_ Motivation M_13_ Feeling heard, understood, supported M_14_ Increased self-esteem M_15_ Trust	O_4_ Acceptability (of delivery) O_5_ Integration and reach
		Illustrative extract: Participants were allowed to form their own groups made up of people with whom they felt comfortable. Ground rules were established at the beginning of each session. The ground rules included being respectful, listening, allowing the space to be free from judgement, maintaining confidentiality and having the freedom to leave the room if needed.^84^ (p. 390)				
	ICAMOC_9_	I_9_ Engaging via peer advocates	C_3_ Workforce capacity C_5_ Values, beliefs, concerns C_6_ Existing relationships C_8_ PPDW with complex needs C_10_ Prison security and regime	A_4_ PPDW	M_9_ Sense of identification (with peers) M_13_ Feeling heard, understood, supported M_15_ Trust	O_4_ Acceptability (of delivery) O_5_ Integration and reach
		Illustrative extract: Peer support provided by The Hepatitis C Trust which was vital for ensuring treatment adherence and following patients on treatment either to new prisons after transfer, or into the community after release. The peers have a lived experience of HCV and incarceration, meaning they are more trusted by, and can better communicate with, residents.^95^ (p. 7)				
Sustaining: evaluating, adapting	ICAMOC_10_	I_10_ Evaluation-adaptation cycles	C_3_ Workforce capacity C_9_ Availability of resources C_13_ Short sentences	A_1_ Implementation teams	M_1_ Perceived needs (of PPDW /deliverers) M_3_ Perceived utility of intervention M_7_ Clarity, reduced uncertainty	O_2_ Appropriateness O_7_ Fidelity O_8_ Sustainability
		Illustrative extract: Ongoing monitoring can assist in the identification of time points or phases where fidelity lapses occur and result in efforts to recalibrate team performance. In HIV-STIC, fidelity monitoring served to bring problems to the attention of researchers, coaches and teams, enabling corrective action.^74^ (p. 199)				

GC/CT, Neisseria gonorrhoeae and Chlamydia trachomatis; HCV, hepatitis C virus; HIV-STIC, HIV Services and Treatment Implementation in Corrections; ICAMOC, Intervention-Context-Actor-Mechanism-Outcomes configuration; NCDOC, North Carolina Department of Corrections; NIATx, Network for the Improvement of Addiction Treatment; PPDW, people in prisons that detain women; STI, sexually transmitted infection.

#### Planning

##### Teaming

Prisons were challenging settings to implement interventions. Prison regimes (ie, routines and activities), regulations and lockdowns were restrictive, disruptive and unpredictable (C_10_).^74 77 85 89 93 98^ Recruitment challenges and competing priorities constrained workforce capacity, and facilities, equipment and funding were limited and well-guarded (C_3,4,9_).^74 77 82 93^ Accessing resources required teaming (I_1,2_), that is, the collaborative effort of multiple stakeholders (hereafter ‘implementation teams’) (A_1_) with the required ‘expertise and authority’^74^ (p. 189), but whose priorities may diverge, like the implicit tension of care versus control between healthcare and prison staff.^7577 82 83 89 93^,^95^

###### ICAMOC_1_—sharing information to facilitate teaming

Analysis suggests that teaming enables implementation. This requires actors to buy-in (O_1_), which is influenced by prior knowledge, organisational culture, personal values, capacity and competing priorities (C_1,2,5,3,4_). If information is shared (I_1_) to engage implementation teams and decision-makers (A_1,2_), then buy-in is more likely, because it increases their perception of PPDW’s health needs, the risks of inaction and the relative advantage of intervention over the status quo (M_1,2,3_).^74 77 86 89 93^ Information sharing throughout implementation maintains and expands buy-in, facilitating adoption and appropriateness (O_1,2_), and should include prison staff (A_3_) as potential facilitators of access.^78 84 86 92 93 95 98^

###### ICAMOC_2_—effective team leadership

If implementation leaders provide a clear vision and practical direction (I_2_) for team members (A_1_), then the latter are more likely to prioritise implementation alongside other commitments (C_4_), increasing feasibility and adoption (O_3,1_), because they are empowered by a shared sense of purpose, agency and clarity of role (M_4,5,6,7_).^74 77 86 94^ Effective leadership is moderated by the regularity of team meetings, quality and frequency of communication and the leader’s level of commitment (C_7,3,5_).^74 76 77 93^ Leadership can be formal or informal and is facilitated by existing relationships and influence (C_6_).^74 75 78 81^

### Assessing needs and capacity

While teaming overcame some barriers, a prison’s capacity for implementation remained a significant influencing factor. Analysis indicates that implementation strategies to add, modify or leverage resources benefitted from prior assessment of needs and potential capacity (I_3_).^74 77^ Resulting outputs were more appropriate, acceptable and feasible (O_2,4,3_) for PPDW, people delivering the intervention (hereafter ‘deliverers’) and decision-makers (A_4,5,2_) alike. Studies used various formative research methods, including focus groups, surveys, site visits, stakeholder consultations and data analysis.^74 75 78 79 85 95^

#### ICAMO_3_—assessing needs and context through formative research

If formative research is carried out (I_3_) to assess the needs (C_1,3,5,8_) of PPDW and deliverers (A_4,5_) and the context for implementation (C_3,9,10,11_), then implementation teams (A_1_) can tailor interventions and plan implementation to be appropriate, acceptable and feasible (O_2,4,3_), because they perceive the needs and preferences of those involved and uncertainty about capacity is reduced (M_1,7_). In studies, the needs of PPDW (A_4_) included learning and communication, neurodiversity, literacy, language and inclusivity alongside relevant values, beliefs and behaviours (C_1,5,8_).^75 79 84 93^ Interventions such as SHE^75 78^ assessed the needs of PPDW holistically within wider determinants (C_8_) to good effect.^76 79^ Capacity factors included facilities, equipment, digital resources and information systems and the limitations of security protocols and prison regimes (C_9,10_).^77 79 83 93 98^ Workforce capacity influenced implementation, encompassing skills/specialisms, training needs, time, engagement and concerns such as fearing needlestick injury (C_1,3,5_).^73 74 77 78 83 84 93 98^ Heterogeneity between prisons and non-adherence to national recommendations was observed (C_11,12_).^82 83 90 93 98^

### Tailoring and planning

Building on formative research to tailor interventions and plan implementation increased appropriateness, acceptability and feasibility (O_2,4,3_). Studies found that, due to PPDW’s (A_4_) experiences of trauma, stigma and lack of autonomy (C_8,5,10_), designing empowering interventions that enabled informed decision-making motivated health-promoting behaviours (M_4,6,8_).^78 79 85 87 88 93^ Furthermore, selecting safe, streamlined, timely innovations/programmes increased access to results/care, as many PPDW serve short sentences (C_13_).^75 92 94 95 98^

#### ICOMOC_4_—tailoring evidence-based interventions to meet the needs of people in prisons that detain women

If evidence-based frameworks and interventions are adopted/adapted (I_4_)^73^,^7985 86^ to design interventions which meet the needs (C_1,5,8,10_) of PPDW (A_4_), then implementation will be more acceptable and appropriate (O_4,2_), because PPDW identify with the content and feel empowered and able to make health-promoting decisions (M_9,4,6,8_). Engaging PPDW in tailoring activities was found to assist this process.^78 85 87^

#### ICOMOC_5_—planning integrated, universal services

Rapid, intensive interventions can be resource-heavy and lack sustained impact.^92 94^ Conversely, if strategies integrate interventions (I_5_) into existing processes (C_11_), then feasibility, cost-effectiveness and engagement (O_3,6,5_) improve, particularly when nudged (M_10_) by universal, opt-out offers.^80^,^8486 94 95 98^ Furthermore, universal offers normalise (M_11_) screening for PPDW (A_4_) by reducing stigma and increasing exposure on recidivism (C_5,13_) and for deliverers (A_5_) by reducing the burden of risk assessment (contingent on time, knowledge, priorities and preconceptions (C_3,1,4,5_)), decreasing uncertainty (M_7_) and increasing asymptomatic disease detection.^80^,^8294 98^ Analysis suggests integration with reception screening increases access, befitting short sentences and prison regimes (C_11,13,10_), but potentially overwhelms PPDW (A_4_) with competing priorities and procedures (C_4_).^7883 84 86 87 93^,^95 98^ Strategies using in-reach, telemedicine or single-point-of-access models may reduce structural barriers but require careful planning.^91 96 98^ While some implementations leverage existing skills and assets (C_1,3,9_),^73 93 95^ sufficient time for workforce recruitment, training, information system development and funding applications is required.^78 79 84 85 87 91 94^

### Doing

#### Implementing

Even when implementation had adequate planning and stakeholder support, prisons remained unpredictable, complex systems for action.^74 79^

##### ICAMOC_6_—piloting and testing implementation

Given resource constraints and workforce capacity (C_9,3_), analysis suggests that if interventions are initially piloted (I_6_), then implementation teams, deliverers, decision-makers and PPDW (A_1,5,2,4_) can perceive its utility and gain clarity about its effectiveness (M_3,7_), informing iterative refinement to ensure feasibility and acceptability, thus enabling fidelity and wider adoption (O_3,4,7,1_).^74^,^7679 84 85^ In the studies, pilots ranged from several days^75^ to several months,^74^ depending on intervention type. After piloting, incremental adoption improved integration and sustainability (O_5,8_), reducing the confusion (M_7_) caused by multiple short-lived interventions.^88 92 93^

##### ICAMOC_7_—standardising and supporting implementation

If introductory training and standardised operating procedures (SOPs) (I_7_) are provided to deliverers (A_5_), then adoption, fidelity and cost-effectiveness improve (O_1,7,6_), within workforce capacity and skill constraints, and particularly when interventions are complex (C_3,1,14_), because they gain clarity about the process, reducing uncertainty and improving self-efficacy (M_7,12_).^74 75 77 82 86^ This is moderated by ongoing technical, clinical, administrative and emotional support, such as helping peers cope with difficult conversations or providing guidance on data utilisation.^74 85 93 95^ Analysis suggests that SOPs consider the whole intervention pathway to aid responsiveness, including linkages to care, escalation/referral routes and contingency plans (C_11,8,10_).^74 75 79 83^

### Engaging

While the implementation processes discussed thus far highlight the overall importance of engagement, this section focuses specifically on what works to engage PPDW in interventions.

Studies concurred that for PPDW (A_4_), lifetime trauma was common and imprisonment was distressing and disempowering (C_8,10_).^75 84 93^ Previous negative experiences fostered deep-seated mistrust of healthcare deliverers, and fear of stigma, judgement, tests and disease were common (C_5,6_).^7578 86 93 96^,^98^ PPDW often felt misunderstood and disinclined to seek help or disclose personal information (M_13,8_).^93 97^ Fasula *et al*^79^ highlight the correlation between a PPDW’s self-esteem and their motivation to adopt health-promoting behaviours (M_14,8_).

#### ICAMOC_8_—trauma-informed engagement

If deliverers (A_5_) adopt empathetic, trauma-informed approaches throughout the intervention pathway (I_8_), then PPDW (A_4_) engage more (O_5_), because they feel heard, understood and supported, which builds self-esteem, agency and trust, increasing empowerment and motivation (M_13,14,6,15,4,8_) and improving acceptability (O_4_).^75 78 79 84 85 93 96 97^ Trauma-informed methods facilitate informed decision-making through affirmations, tailored education, information-sharing, signposting, expectation-setting and offering choices.^78 85 87 88 93^ Group modalities promote emotional safety, decreasing power differentials and fostering social support.^75 84 85^ Flyers, posters, workshops and word-of-mouth can raise awareness, and verbal communication helps overcome literacy barriers.^73 75 78 88^ Optimising coverage should be weighed against giving sufficient time for decision-making to avoid coercion, particularly for long-acting contraception.^88 89^

#### ICAMOC_9_—engaging via peer advocates

If advocacy, particularly by peers (I_9_), is provided, then acceptability, integration and reach increase (O_4,5_), because PPDW (A_4_) identify with and trust them (M_9,15_) and because increased opportunities to engage within the prison regime (eg, housing blocks, reception and post-release) (C_10_) help them feel heard and supported (M_13_).^73 93 95 98^ Analysis suggests that, with appropriate training and support, peers find advocacy roles affirming and empowering, becoming champions for the intervention and augmenting workforce capacity (C_3_).^73 93^

### Sustaining

#### Evaluating and adapting

Although the CFIR distinguishes between *reflection and evaluation,* characterised by the collection and analysis of information and *adaptation* of the intervention or context, they are combined here as study data support adopting an ongoing, iterative process for improved sustainability and relevance.

##### ICAMOC_10_—evaluation-adaptation cycles

If evaluation-adaptation cycles are employed (I_10_), based on relevant information and feedback,^74 79^ then appropriateness, fidelity and sustainability improve (O_2,7,8_), because implementation teams (A_1_) gain clarity over the utility of the intervention and whether implementation meets the needs of deliverers and recipients (M_7,3,1_) within the context of available resources (C_3,9_).^74 79 83 85^ Studies sought feedback from various stakeholders, including peers, deliverers and PPDW, although short sentences and abrupt cessation (C_13,9_) of funding sometimes hindered collection.^76 78 79 81 87 93^ Focus groups, surveys, information systems and evaluation tools were used, but data availability and its effective utilisation were limited and the importance of impartiality and transparency were stressed.^74 77 79 83 85 87 90 93 95^ Pankow *et al*^74^ recommend ongoing process evaluation to ensure fidelity (O_7_), while elsewhere positive feedback was noted as rewarding for deliverers.^93^

### Refined programme theory

**Figure 3:**
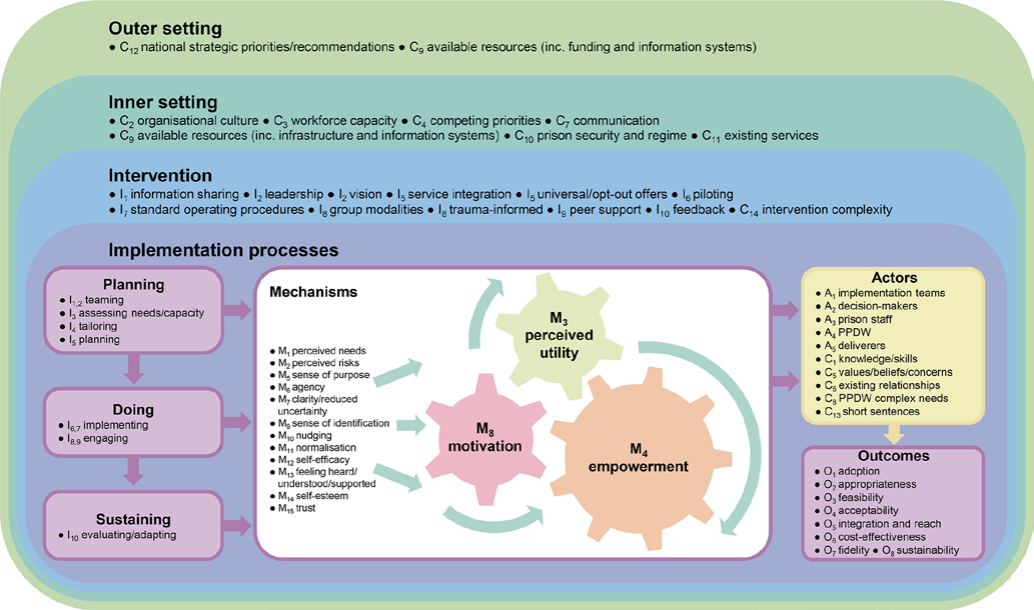
Refined programme theory. PPDW, people in prisons that detain women.

Explanatory factors from cross-case ICAMOCs were used to refine the PT (see figure 3). The mechanisms identified were grouped into three higher level mechanisms found to be relevant to all actors, namely, perceived utility of intervention, motivation to implement and empowerment to take action.

## Discussion

### Summary of findings

We identified three overarching themes. First, workforce capacity is a significant constraining factor when implementing women’s sexual and reproductive health interventions in prisons. Recruitment and retention are ongoing issues for prison staff and deliverers, for whom core duties are reactive and challenging. Deliverers can feel uncertain and lack time and resources to manage competing priorities effectively. Analysis indicates effective leadership, training, ongoing support and SOPs enable deliverers to perceive the utility of implementing interventions and gain clarity about their role. Universal and opt-out offers reduce reliance on deliverers’ time and inclination to risk assess and on a PPDW’s willingness to disclose medical or behavioural history, thereby increasing coverage. Mobilising peers from the prison population and/or external organisations can enhance capacity, providing an affirming experience for the peer when adequately trained and supported.^73 93^ Actor types involved in planning should seek to empower the workforce through training, support and augmentation to make implementation more feasible and acceptable.

Second, the persistence of competing priorities hinders engagement across all actor types. Decision-makers and prison staff work within strict protocols to safeguard the workforce and PPDW, prioritising security. Healthcare deliverers necessarily prioritise high-volume urgent medical needs within resource constraints. PPDW do not often prioritise their sexual and reproductive health, particularly at reception when coping with, for example, mother-child separation, substance withdrawal and situational distress—as Hilkey postulates, Maslow’s theory of human motivation places such pressing physiological and psychological needs above health-promotion in prisons.^99^ This is evident in the dominance of HCV and HIV studies in this review which, while included here as STIs, may be prioritised over other women’s sexual and reproductive health issues (like menstruation or menopause) due to their association with intravenous drug use. Education and information-sharing to increase understanding of women’s sexual and reproductive health needs and associated risks enable actors to perceive the utility of intervention, increasing their motivation for intervention. This aligns with the concepts of perceived susceptibility, severity and benefits central to the Health Belief Model.^100^ Sustained, meaningful efforts to raise awareness about women’s sexual and reproductive health in prisons are needed. Several studies demonstrated that long-term research partnerships can facilitate this and help to expand the evidence base.^73 74 78^

Lastly, adopting a trauma-informed approach throughout implementation activities is an important enabling factor. PPDW often have significant lifetime trauma resulting in low self-esteem, low self-efficacy and pervading fear and mistrust. People who use drugs and sex workers can feel stigmatised and misunderstood, and prisons fundamentally and profoundly restrict autonomy. The resulting sense of disempowerment is worsened when deliverers seem judgemental, disinterested or dismissive. In contrast, empathetic interactions in which PPDW feel emotionally safe to talk openly and feel heard and understood are empowering and normalising. Providing education and opportunities for PPDW to make informed, uncoerced decisions about their health aligns with the self-determination theory of motivation.^101^ Furthermore, group activities which promote respect, equality and acceptance offer much-needed opportunities for PPDW to build social support. Advocate roles, including peer-based programmes, can provide trustworthy support, bridging the gap between deliverer and recipient, and should be considered in all prison settings. There is increasing evidence to support trauma-informed care in prisons, and on-going investment in workforce training and the adoption of trauma-informed care throughout the prison estate, or network, is recommended.^102^,^104^

### Conclusions and recommendations

The Chief Medical Officer’s 2025 annual report on the physical health of people in England’s prisons recommends prioritising health prevention interventions to tackle inequities within the system and improve health outcomes.^5^ Recognising the complex challenges of implementation in custodial settings and the unmet needs particular to PPDW, the objectives of this review were to explore what works for whom, how and why, when implementing women’s sexual and reproductive health interventions in prisons. This involved exploring the barriers and facilitators to implementation described in primary studies to generate recommendations for policymakers. The CFIR informed the IPT, which was tested and refined through realist synthesis to refine an explanatory PT (figure 3). Three main mechanisms of perceived utility of intervention, motivation and empowerment were identified and informed the following recommendations for policymakers:

- Build a resilient, empowered delivery workforce through training, support and augmentation.
- Invest in research partnerships to increase awareness and understanding of women’s sexual and reproductive health in prisons and address the evidence gap.
- Promote trauma-informed methods to develop and deliver women’s sexual and reproductive health services throughout the prison estate.

### Strengths and limitations

A strength of this review is that it synthesises evidence from a range of sources to theorise what works when implementing women’s health interventions in prisons. This addresses a significant gap in the literature and could form the basis for further research through case studies or as new studies emerge. While including unpublished literature may lower publication bias, only studies from high-income countries published in English since 2010 were included, which was a limitation of the search strategy. Furthermore, the predominance of North American studies (n=19) may limit generalisability to England, particularly given policy differences in criminal justice and healthcare systems.

None of the studies focused specifically on menopause, menstruation or pregnancy testing, despite searches including these terms. In addition, use of the WHH specification and prison policy/guidance to define women’s sexual and reproductive health domains may have excluded relevant alternatives, such as abortion. The search strategy could have been further strengthened by including specific search terms for non-binary and gender diverse participants. While Pawson *et al*^57^ advise pragmatism to balance theoretical saturation with the availability of studies and time, further purposive sampling could have been undertaken. These limitations could have been addressed, and the search strategy strengthened, through stakeholder or advisory group involvement.

## Supplementary material

10.1136/bmjopen-2025-113940 online supplemental file 1

## Data Availability

Data are available upon reasonable request.

## References

[1] Stürup-Toft S, O’Moore EJ, Plugge EH (2018). Looking behind the bars: emerging health issues for people in prison. Br Med Bull.

[2] World Health Organisation (2022). Cancer and cardiovascular health inequities in prison settings: a rapid literature review. Copenhagen. https://iris.who.int/bitstream/handle/10665/360079/WHO-EURO-2022-5814-45579-65357-eng.pdf?sequence=1.

[3] Greene M, Ahalt C, Stijacic-Cenzer I (2018). Older adults in jail: high rates and early onset of geriatric conditions. Health Justice.

[4] Enggist S, Møller L, Galea G (2014). Prisons and *h*ealth.

[5] Whitty C (2025). The health of people in prison, on probation and in the secure NHS estate in England. London. https://www.gov.uk/government/publications/the-health-of-people-in-prison-on-probation-and-in-the-secure-nhs-estate-in-england.

[6] NHS England (2023). A review of health and social care in women’s prisons, 22. https://www.england.nhs.uk/long-read/a-review-of-health-and-social-care-in-womens-prisons/#8-appendix-two-profile-of-women-in-prison.

[7] Friestad C, Haukvik UK, Johnsen B (2023). Prevalence and characteristics of mental and physical disorders among female prisoners: a mixed-methods systematic review. IJPH.

[8] Massoglia M, Pare P-P, Schnittker J (2014). The relationship between incarceration and premature adult mortality: Gender specific evidence. Soc Sci Res.

[9] Balis AF, Greifinger RB (2022). Public health behind bars.

[10] Emerson A, Lipnicky A, Schuster B (2022). Physical health programs and interventions with women during incarceration: a scoping review. IJPH.

[11] Fazel S, Bains P, Doll H (2006). Substance abuse and dependence in prisoners: a systematic review. Addiction.

[12] Escobar N, Plugge E (2020). Prevalence of human papillomavirus infection, cervical intraepithelial neoplasia and cervical cancer in imprisoned women worldwide: a systematic review and meta-analysis. J Epidemiol Community Health.

[13] Brousseau EC, Ahn S, Matteson KA (2019). Cervical Cancer Screening Access, Outcomes, and Prevalence of Dysplasia in Correctional Facilities: A Systematic Review. J Womens Health.

[14] Spaulding AC, Rabeeah Z, Del Mar González-Montalvo M (2022). Prevalence and Management of Sexually Transmitted Infections in Correctional Settings: A Systematic Review. Clin Infect Dis.

[15] Gharagozloo M, Moridi M, Alimardi M (2025). Reproductive health needs of incarcerated women in developed countries: a mixed-method systematic review. Eur J Med Res.

[16] Binswanger IA, Mueller S, Clark CB (2011). Risk Factors for Cervical Cancer in Criminal Justice Settings. J Womens Health.

[17] Davies EA, Lüchtenborg M, Maree Hunter R (2025). Cancer in English prisons: a mixed-methods study of diagnosis, treatment, care costs and patient and staff experiences. *Health Soc Care Deliv Res*.

[18] Hunter RM, Huynh J, Lüchtenborg M (2024). Does the cost of cancer care for people in prison differ from those in the general population? Analysis of matched English cancer registry and hospital records. EClinicalMedicine.

[19] United Kingdom. Department of Health & Social Care (2022). Women’s health strategy for England. https://assets.publishing.service.gov.uk/media/6308dbe1e90e0729dcbd7ebb/Womens-Health-Strategy-England-print.pdf.

[20] Daniel K, Bousfield J, Hocking L (2024). Women’s Health Hubs: a rapid mixed-methods evaluation. Health Soc Care Deliv Res.

[21] United Kingdom. Office for Health Improvement & Disparities (2022). Population screening: review of interventions to improve participation among underserved groups. https://www.gov.uk/government/publications/population-screening-improving-participation-in-underserved-groups/population-screening-review-of-interventions-to-improve-participation-among-underserved-groups#results.

[22] McConnon A, Fung K, Lofters A (2019). Colorectal and Breast Cancer Screening Status for People in Ontario Provincial Correctional Facilities. Am J Prev Med.

[23] NHS England (2023). Specification 29 section 7A: public health services for children and adults in secure and detained settings in England, 2023-2024. https://www.england.nhs.uk/long-read/specification-29-section-7a-public-health-services-for-children-and-adults-in-secure-and-detained-settings-in-england-2023-2024/#annex-a-health-and-justice-indicators-of-performance.

[24] Plugge E, Fitzpatrick R (2004). Factors affecting cervical screening uptake in prisoners. J Med Screen.

[25] Holman D, Salway S, Bell A (2021). Can intersectionality help with understanding and tackling health inequalities? Perspectives of professional stakeholders. Health Res Policy Syst.

[26] Nagami EH, Rich JD, Lee JD, Greifinger RB (2022). Public health behind bars.

[27] Blackaby J, Byrne J, Bellass S (2023). Interventions to improve the implementation of evidence-based healthcare in prisons: a scoping review. Health Justice.

[28] World Health Organisation (2021). The WHO prison health framework: a framework for assessment of prison health system performance. Copenhagen. https://iris.who.int/bitstream/handle/10665/344561/9789289055482-eng.pdf?sequence=3.

[29] Woodall J, Dixey R, South J (2014). Control and choice in English prisons: developing health-promoting prisons. Health Promot Int.

[30] Penal Reform International (2021). Guidance document on the Bangkok rules: implementing the united nations rules on the treatment of women prisoners and non-custodial measures for women offenders.

[31] Kouyoumdjian FG, McIsaac KE, Liauw J (2015). A Systematic Review of Randomized Controlled Trials of Interventions to Improve the Health of Persons During Imprisonment and in the Year After Release. Am J Public Health.

[32] National Institute for Health and Care Research (2024). 23/146 interventions to support women in prison or post-release v1.1. https://www.nihr.ac.uk/23146-interventions-support-women-prison-or-post-release.

[33] Paynter M, Heggie C, McKibbon S (2022). Sexual and Reproductive Health Outcomes among Incarcerated Women in Canada: A Scoping Review. Can J Nurs Res.

[34] Wong J, Carpenter L, Bunn J (2020). P52 Family planning services for women incarcerated in the US: A scoping review. Contraception.

[35] Murray C, Campbell E, Burns D (2025). Women’s experiences of needing abortion care whilst incarcerated: a systematic review of the international literature. Cult Health Sex.

[36] Peart MS, Knittel AK (2020). Contraception need and available services among incarcerated women in the United States: a systematic review. Contracept Reprod Med.

[37] Hoff E, Adams ZM, Grimshaw A (2021). Reproductive Life Goals: A Systematic Review of Pregnancy Planning Intentions, Needs, and Interventions Among Women Involved in U.S. Criminal Justice Systems. J Womens Health.

[38] Hamilton A, Shin S, Taggart T (2020). HIV testing barriers and intervention strategies among men, transgender women, female sex workers and incarcerated persons in the Caribbean: a systematic review. Sex Transm Infect.

[39] Staley H, Shiraz A, Shreeve N (2021). Interventions targeted at women to encourage the uptake of cervical screening. Cochrane Database Syst Rev.

[40] Baron RC, Melillo S, Rimer BK (2010). Intervention to increase recommendation and delivery of screening for breast, cervical, and colorectal cancers by healthcare providers a systematic review of provider reminders. Am J Prev Med.

[41] Bashirian S, Mohammadi Y, Barati M (2020). Effectiveness of the Theory-Based Educational Interventions on Screening of Breast Cancer in Women: A Systematic Review and Meta-Analysis. Int Q Community Health Educ.

[42] Ferrari A, Jael Herrera D, Van De Veerdonk W (2025). Advancing Mammographic Screening Among Underserved Groups: A Systematic Review and Meta-Analysis of Intervention Strategies to Increase Breast Cancer Screening Uptake. Public Health Rev.

[43] Qian J, Sun S, Wang M (2023). The effect of exercise intervention on improving sleep in menopausal women: a systematic review and meta-analysis. Front Med.

[44] Proctor EK, Landsverk J, Aarons G (2009). Implementation Research in Mental Health Services: an Emerging Science with Conceptual, Methodological, and Training challenges. Adm Policy Ment Health.

[45] Lobb R, Colditz GA (2013). Implementation Science and Its Application to Population Health. Annu Rev Public Health.

[46] Sturge G (2024). UK prison population statistics. London. https://researchbriefings.files.parliament.uk/documents/SN04334/SN04334.pdf.

[47] Zielinski MJ, Allison MK, Brinkley-Rubinstein L (2020). Making change happen in criminal justice settings: leveraging implementation science to improve mental health care. Health Justice.

[48] Skivington K, Matthews L, Simpson SA (2021). A new framework for developing and evaluating complex interventions: update of Medical Research Council guidance. BMJ.

[49] Pawson R, Greenhalgh T, Harvey G (2005). Realist review - a new method of systematic review designed for complex policy interventions. J Health Serv Res Policy.

[50] Shearn K, Allmark P, Piercy H (2017). Building Realist Program Theory for Large Complex and Messy Interventions. Int J Qual Methods.

[51] Lacouture A, Breton E, Guichard A (2015). The concept of mechanism from a realist approach: a scoping review to facilitate its operationalization in public health program evaluation. Implement Sci.

[52] Dalkin SM, Greenhalgh J, Jones D (2015). What’s in a mechanism? Development of a key concept in realist evaluation. Implement Sci.

[53] Mukumbang FC, Kabongo EM, Eastwood JG (2021). Examining the Application of Retroductive Theorizing in Realist-Informed Studies. Int J Qual Methods.

[54] McEwan K, Girling M, Bate A (2024). ‘For Want of a Nail’: developing a transparent approach to retroduction and early initial programme theory development in a realist evaluation of community end of life care services. Int J Soc Res Methodol.

[55] Rycroft-Malone J, McCormack B, Hutchinson AM (2012). Realist synthesis: illustrating the method for implementation research. Implement Sci.

[56] Flynn R, Schick-Makaroff K, Levay A (2020). Developing an Initial Program Theory to Explain How Patient-Reported Outcomes Are Used in Health Care Settings: Methodological Process and Lessons Learned. Int J Qual Methods.

[57] Pawson R (2006). Evidence-*based pol*icy.

[58] Wong G, Greenhalgh T, Westhorp G (2013). RAMESES publication standards: realist syntheses. BMC Med.

[59] Marchal B, Kegels G, Van Belle S, Emmel N, Greenhalgh J, Manzano A (2018). Doing realist research.

[60] Buck D, Mulligan LD, Lennox C (2025). Developing an initial programme theory for a model of social care in prisons and on release (empowered together): A realist synthesis approach. Med Sci Law.

[61] Mukumbang FC, Marchal B, Van Belle S (2018). Unearthing how, why, for whom and under what health system conditions the antiretroviral treatment adherence club intervention in South Africa works: A realist theory refining approach. BMC Health Serv Res.

[62] De Weger E, Van Vooren NJE, Wong G (2020). What’s in a Realist Configuration? Deciding Which Causal Configurations to Use, How, and Why. Int J Qual Methods.

[63] Abejirinde I-O, Ilozumba O, Marchal B (2018). Mobile health and the performance of maternal health care workers in low- and middle-income countries: A realist review. Int J Care Coord.

[64] United Kingdom. Department of Health & Social Care (2024). Women’s health hubs: core specification. https://www.gov.uk/government/publications/womens-health-hubs-information-and-guidance/womens-health-hubs-core-specification.

[65] National Institute for Health and Care Excellence (2016). Physical health of people in prison. United Kingdom. https://www.nice.org.uk/guidance/ng57.

[66] British Association of Sexual Health and HIV (2023). Standards for the management of sexual health in UK prisons. Macclesfield. https://www.bashh.org/_userfiles/pages/files/resources/3079_prison_standards_bashh_1_final.pdf.

[67] Damschroder LJ, Reardon CM, Widerquist MAO (2022). The updated Consolidated Framework for Implementation Research based on user feedback. Implement Sci.

[68] Proctor E, Silmere H, Raghavan R (2011). Outcomes for implementation research: conceptual distinctions, measurement challenges, and research agenda. *Adm Policy Ment Health*.

[69] The World Bank (2025). World bank country and lending groups. https://datahelpdesk.worldbank.org/knowledgebase/articles/906519-world-bank-country-and-lending-groups.

[70] Dada S, Dalkin S, Gilmore B (2023). Applying and reporting relevance, richness and rigour in realist evidence appraisals: Advancing key concepts in realist reviews. Res Synth Methods.

[71] Ho L, Limpaecher A (2025). Delve. https://delvetool.com/.

[72] Pelz W (2025). Research methods for the social sciences. https://socialsci.libretexts.org/Bookshelves/Sociology/Introduction_to_Research_Methods/Research_Methods_for_the_Social_Sciences_(Pelz)/01%3A_Chapters/1.13%3A_Chapter_13_Qualitative_Analysis.

[73] Thornton K, Sedillo ML, Kalishman S (2018). The New Mexico Peer Education Project: Filling a Critical Gap in HCV Prison Education. J Health Care Poor Underserved.

[74] Pankow J, Willett J, Yang Y (2018). Evaluating Fidelity to a Modified NIATx Process Improvement Strategy for Improving HIV Services in Correctional Facilities. J Behav Health Serv Res.

[75] Kennedy P, Ratnaparkhi R, Lee J (2023). Case example of a jail-based cancer prevention clinical trial: Social determinants of health framework, novel experimental design, and retention strategies to facilitate long-term follow-up of clinical trial participants. J Clin Trans Sci.

[76] Kelly PJ, Driscoll D, Lipnicky A (2022). Developing a Cancer Prevention Health Education Resource: a Primer of Process and Evaluation. J Canc Educ.

[77] Emerson A, Allison M, Kelly PJ (2020). Barriers and facilitators of implementing a collaborative HPV vaccine program in an incarcerated population: A case study. Vaccine.

[78] Ramaswamy M, Simmons R, Kelly PJ (2015). The development of a brief jail-based cervical health promotion intervention. Health Promot Pract.

[79] Fasula AM, Fogel CI, Gelaude D (2013). Project power: Adapting an evidence-based HIV/STI prevention intervention for incarcerated women. AIDS Educ Prev.

[80] Dang CM, Pao J, Taherzadeh D (2021). Paired Testing of Sexually Transmitted Infections With Urine Pregnancy Tests in Incarcerated Women. Sex Transm Dis.

[81] Cocoros N, Nettle E, Church D (2014). Screening for Hepatitis C as a Prevention Enhancement (SHAPE) for HIV: an integration pilot initiative in a Massachusetts County correctional facility. Public Health Rep.

[82] Cole J, Hotton A, Zawitz C (2014). Opt-out screening for Chlamydia trachomatis and Neisseria gonorrhoeae in female detainees at Cook County jail in Chicago, IL. Sex Transm Dis.

[83] Harmon JL, Dhaliwal SK, Burghardt NO (2020). Routine Screening in a California Jail. Public Health Rep.

[84] Holton LM, Hampton MD (2023). Implementing High-Intensity, Trauma-Informed Sexual Risk Reduction in Women Involved in the Criminal Legal System. J Correct Health Care.

[85] Johnson JE, Peabody ME, Wechsberg WM (2015). Feasibility of an HIV/STI Risk-Reduction Program for Incarcerated Women Who Have Experienced Interpersonal Violence. *J Interpers Violence*.

[86] Murphy M, Rogers BG, Ames E (2024). Implementing Preexposure Prophylaxis for HIV Prevention in a Statewide Correctional System in the United States. *Public Health Rep*.

[87] McNeely CA, Hutson S, Sturdivant TL (2019). Expanding Contraceptive Access for Women With Substance Use Disorders: Partnerships Between Public Health Departments and County Jails. J Public Health Manag Pract.

[88] Sufrin C, Oxnard T, Goldenson J (2015). Long‐Acting Reversible Contraceptives for Incarcerated Women: Feasibility and Safety of On‐Site Provision. Perspect Sexual Reproductive.

[89] Sufrin C, Baird S, Clarke J (2017). Family planning services for incarcerated women: models for filling an unmet need. IJPH.

[90] Winter RJ, White B, Kinner SA (2016). A nurse‐led intervention improved blood‐borne virus testing and vaccination in Victorian prisons. Aust N Z J Public Health.

[91] Hesse S, Williamson K, Bonney D (2023). Cancer screening in prisons: lessons for health providers. Aust J Prim Health.

[92] Wallis C, O’Flynn M, Fenech M (2023). Hepatitis C virus point-of-care RNA testing: Experience from screening an entire high-security Australian prison population over 3 days. Aust N Z J Public Health.

[93] Against Violence & Abuse (2019). An evaluation of women in prison’s health matters project. London. https://womeninprison.org.uk/media/downloads/health-matters-final-evaluation-report.pdf.

[94] Phaw NA, Thant AM, Thompson C (2025). Prospective evaluation of the impact of repeated whole prison testing for hepatitis C. BMJ Open Gastroenterol.

[95] Mongale E, Allen S, Brew I (2024). Development and optimisation of a reception testing protocol designed to eliminate HCV in the UK prison population. JHEP Rep.

[96] Besney JD, Angel C, Pyne D (2018). Addressing Women’s Unmet Health Care Needs in a Canadian Remand Center: Catalyst for Improved Health?. J Correct Health Care.

[97] Paynter M, Heggie C, McLeod A (2025). The experiences of family planning health professionals providing care to incarcerated patients: a qualitative study. BMC Pregnancy Childbirth.

[98] Crowley D, Van Hout MC, Murphy C (2019). Hepatitis C virus screening and treatment in Irish prisons from nurse managers’ perspectives - a qualitative exploration. BMC Nurs.

[99] Hilkey JH (1988). A Theoretical Model for Assessment of Delivery of Mental Health Services in the Correctional Facility. Psychiatr Ann.

[100] National Cancer Institute (2005). Theory at a glance: a guide for health promotion practice.

[101] Hagger MS, Hankonen N, Chatzisarantis NLD (2020). The handbook of behavior change.

[102] Gaber J, Scallan E, Kouyoumdjian FG (2025). Understanding Trauma-Informed Care in Correctional Facilities: A Scoping Review. *J Correct Health Care*.

[103] Malik N, Facer-Irwin E, Dickson H (2023). The Effectiveness of Trauma-Focused Interventions in Prison Settings: A Systematic Review and Meta-Analysis. Trauma Violence Abuse.

[104] Harner H, Burgess AW (2011). Using a Trauma‐Informed Framework to Care for Incarcerated Women. J Obstet Gynecol Neonatal Nurs.

[105] Smeets RGM, Hertroijs DFL, Mukumbang FC (2022). First Things First: How to Elicit the Initial Program Theory for a Realist Evaluation of Complex Integrated Care Programs. Milbank Quarterly.

[106] Westhorp G, Emmel N, Greenhalgh J, Manzano A (2018). Doing realist research.

[107] Cambon L, Terral P, Alla F (2019). From intervention to interventional system: towards greater theorization in population health intervention research. BMC Public Health.

[108] Pearsons A, Neubeck L, Hendriks JM (2023). Justification, rationale and methodological approaches to realist reviews. Eur J Cardiovasc Nurs.

