# Supplementary material for: Implementing women’s sexual and reproductive health interventions in prisons: a realist review
Source: BMJ Open. 2026 Jun 22;16(6):e113940. doi: 10.1136/bmjopen-2025-113940 (PMC13288683; doi:10.1136/bmjopen-2025-113940)
Supplement: online supplemental file 1 [file bmjopen-16-6-s001.docx]

**SUPPLEMENTARY FILE 1**

# Conceptual framework of initial programme theory (IPT) for implementing women’s health interventions in prisons:


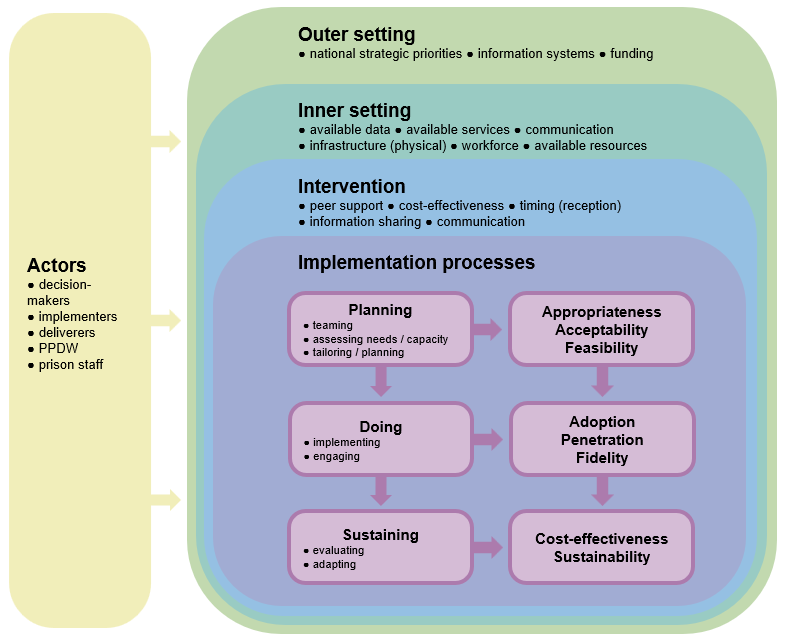


# Main scientific database search terms

Ovid MEDLINE, Global Health, APA PsycINFO, CINAHL and Embase were searched using the following terms. MeSH terms (shown in **bold**) were changed depending on the database being searched – see page 2 for a list of terms per database.

*Population: Location:*

1. **Subject heading search of 'prison'**
2. (prison* OR jail* OR gaol* OR penitentia* OR reformatory OR watch?house* OR remand).ti,ab.
3. ((correction* OR justice OR judicial OR secure OR detention OR custodial OR penal) adj2 (system* OR centre OR center OR facilit* OR setting OR estate OR sentence)).ti,ab.
4. (inmate* OR convict* OR offender* OR criminal* OR felon* OR remand* OR detaine* Or (incarcerat* NOT hernia*)).ti,ab.
5. 1 Or 2 OR 3 OR 4

*Population: Gender:*

1. **Subject heading search of 'women's health'**
2. (Women* OR woman* OR female*).ti,ab.
3. 6 Or 7

*Intervention:*

1. ((Women* OR woman* OR female* OR reproductive OR sexual) ADJ2 (health* OR wellbeing* OR Wellness Or screening)).ti,ab.
2. **Subject heading search of 'cancer screening'**
3. **Subject heading search of 'cervical screening'**
4. (cervical OR cervix OR “pap smear” OR Papanicolaou OR cytology).ti,ab. 13. **Subject heading search of 'breast screening'**
5. (breast OR mammogra*).ti,ab.
6. **Subject heading search of 'menopause' and 'menstruation'**
7. (menopaus* OR menstruat* OR menstrual).ti,ab.
8. **Subject heading search of 'contraceptive'**
9. (contracepti* OR LARC OR IUD OR "intrauterine device*").ti,ab.
10. **Subject heading search of 'sexually transmitted disease', 'HIV', 'HPV,**

**'Hepatitis', 'chlamydia', 'syphilis' and 'gonorrhoea'**

1. (HIV OR “human immunodeficiency virus” OR STI OR STD OR “sexually transmitted” OR HPV OR “human papillomavirus” OR syphilis OR gonorr* OR HBV OR HCV OR hepatitis OR chlamydia).ti,ab.
2. 9 OR 10 OR 11 OR 12 OR 13 OR 14 OR 15 OR 16 OR 17 OR 18 OR 19 OR 20

*Search Limits:*

1. 5 AND 8 AND 21
2. Limit 22 to English language, published in or after 2010

# Database-specific MeSH search terms

| **Database** | **Search** | **Results** |
| --- | --- | --- |
| **Ovid**  **MEDLINE®**  **All** | 1. *incarceration/ OR *prisons/  6. *Women/ OR *Women's Health/   1. *"Early Detection of Cancer"/ OR *Mass Screening/ 2. *papanicolaou test/ OR *vaginal smears/   13. *Breast Neoplasms/ OR *Breast/ Or *Mammography/  15. exp Menopause/ Or exp Menstruation Disturbances/ OR *Menstruation/  17. exp Contraceptive Agents, Female/ OR exp Contraception/ OR exp Contraceptive Devices/  19. exp Sexually Transmitted Diseases/ OR  *Papillomavirus Infections/ OR *HIV Infections/ OR HIV/ OR *HIV Testing/ OR *Syphilis/ OR *Neisseria gonorrhoeae/ OR *Chlamydia Infections/ OR  *Gonorrhea/ OR (*Hepatitis, Viral, Human/ OR  *Hepatitis Viruses/ OR *Hepatitis/ OR *Hepatitis B/ OR  *Hepatitis C/ NOT exp animal/) | 1379 |
| **Ovid Global**  **Health** | 1. Correctional institutions/  6. Women's Health/   1. Screening/ 2. Papanicolaou testing/ OR cervical cancer/   13. Breast cancer/ Or Mammography/  15. Menopause/ Or Menstruation/  17. Contraception/  19. Sexually Transmitted Diseases/ OR Human papillomaviruses/ OR human immunodeficiency viruses/ OR Syphilis/ OR Gonorrhoea/ OR Chlamydia/ OR Hepatitis/ OR Hepatitis B virus/ OR Hepatits C virus/ | 1081 |
| **APA**  **PsycINFO** | 1. Exp Prisons/  6. N/A   1. Screening Tests/ or Intervention/ or Cancer Screening/ or Screening/ 2. Cervix/   13. Breast Neoplasms/ or Mammography/  15. Menopause/ or menstruation/  17. Birth control/ | 1195 |
| **Database** | **Search** | **Results** |
|  | 19. Sexually Transmitted Diseases/ OR HIV Testing/ or exp  HIV/ OR Human Papillomavirus/ OR Gonorrhoea/ OR  Syphilis/ OR Hepatitis/ |  |
| **CINAHL** | 1. (MH “Correctional facilities+”)  6. (MH “Women’s health+”) OR (MH “women’s health services”)   1. (MH “Cancer Screening”) OR (MH “Health Screening”) 2. (MH “Cervical smears”)   13. (MH “Breast Examination”) OR (MH “Mammography”)  15. (MH “Climacteric+”) OR (MH “Menstruation+”) OR (MH  “Menstrual and Perimenopausal Disorders+”)  17. (MH “Contraception+”) OR (MH “Contraceptive agents+”)  19. (MH “Sexually Transmitted Diseases+”) OR (MH  “Hepatitis”) OR (MH “Hepatitis B”) OR (MH “Hepatitis  C”) | 1111 |
| **Ovid**  **EMBASE** | 1. correctional facility/ or correctional health care/ OR prison nursing/  6. exp Women's Health/   1. *Cancer Screening/ OR *Screening/ OR *Mass Screening/ 2. *Uterine cervix cancer/ OR *vagina smear/ OR   *papanicolaou test/  13. *Breast cancer/ Or *Mammography/  15. *menopause/ OR *early menopause/ OR *menopause related disorder/ OR *menstruation disorder/ or menstruation/  17. *oral contraception/ OR *hormonal contraception/ OR *access to contraception/ OR *emergency contraception/ OR *contraception/ OR *long-acting reversible contraception/  19. exp *Sexually Transmitted Disease/ OR *Papillomavirus Infection/ OR *human immunodeficiency virus/ OR *Syphilis/ OR *Syphilis rapid test/ OR *gonorrhea/ OR *Chlamydia rapid test/ OR *Chlamydia/ OR *hepatitis C rapid test/ OR  *hepatitis B/ OR *hepatitis B rapid test/ OR *hepatitis  C/ OR *hepatitis/ | 2526 |

# Inclusion and Exclusion Criteria

| **Domain** | **Inclusion criteria** | **Exclusion criteria** |
| --- | --- | --- |
| **Population(s)** *types/characteristics of participants; ages of participants; health conditions of participants; etc.* | Females currently in detention (initially include immigration detention centres, then review later)  Aged 18+  HIC | Males  Females <18 years old  Those who are no longer in detention at the start of the intervention  LMIC or cannot be determined |
| **Intervention(s)**  *Types/characteristics of interventions; do articles which only have your target intervention as a part of the study "count"?; etc.* | Interventions to include:   - Cervical screening - Breast screening - STI/HIV screening - Menopause - Menstruation - Contraception   Interventions that target multiple programmes will be included as long as they include at least 1 of the above | Interventions that target males  Programmes that are outside national/general population offer for the UK |
| **Outcome(s)**  *Which specific outcome measures "count"? Which ones don't? What about qualitative evidence?* | Acceptability, adoption, appropriateness,  feasibility, fidelity,  implementation cost, coverage and/or sustainability of the intervention implemented | Studies that do not contain any measure or evaluation of implementation outcomes |
| **Study designs**  *Which study types "count"?*  *Which ones don't?* | Any that describe the implementation or evaluation of a relevant  intervention or interventions  Service evaluations | Editorials  Prevalence/incidence studies  Attitudinal studies  Clinical effectiveness studies |
| **Date or language criteria** | Published between 2010 and 2025  English language only  Full text available | Published prior to 2010  Any language other than English  Full Text not available |
