# Supplementary material for: Implementing women’s sexual and reproductive health interventions in prisons: a realist review
Source: BMJ Open. 2026 Jun 22;16(6):e113940. doi: 10.1136/bmjopen-2025-113940 (PMC13288683; doi:10.1136/bmjopen-2025-113940)
Supplement: Supplementary file 2 [file bmjopen-2025-113940.reviewer_comments.pdf]

PEER REVIEW HISTORY

BMJ Open publishes all reviews undertaken for accepted manuscripts. Reviewers are asked to complete a checklist review form and are provided with free text boxes to elaborate on their assessment. These free text comments are reproduced below.

ARTICLE DETAILS

Title (Provisional)

Implementing Women's Sexual and Reproductive Health Interventions in Prisons: A Realist Review

Authors

Rickard, Joy Louise; Melendez-Torres, G.J

VERSION 1 - REVIEW

**Reviewer**	**1**
**Name**	**Paynter, Martha**
**Affiliation**	**University of New Brunswick**
**Date**	**12-Dec-2025**
**COI**	**None**

Comments:

Dear authors,

Thank you for this important study. I appreciate the strength and relevance of the realist synthesis methodology for the complexity of the topic that is sexual and reproductive health care interventions in prisons for women.

Background: very strong, and useful comparison to both men in prison as well as women in general population

Suggest mentioning of gender diversity early on and use of inclusive language throughout

The claim that prison removes people from harmful environments is somewhat problematic (lines 21-24 p. 4); the potential for prisons to offer healthcare (which they are mandated to provide) could be made more clear to be an as-of-yet-unrealized opportunity

Rationale: clear and concise definition of realist synthesis would be suitable at the beginning of this section

Materials and methods: suggest including detail on author and research team involvement here

“Scoping of the literature" was this done formally/informally? What exactly did this entail?

Suggest refining the introductory paragraph of methods section to include sub-headings and explanation of the sequence in which methods were conducted

Search strategy: how were the databases selected?

Who was the “other reviewer” that conducted the 10% secondary screening?

Who conducted data extraction? Analysis? Discussion “in relation to theories and IPT to derive insights"

Regarding women’s sexual and reproductive health domains, I am curious as to why interventions to improve access to abortion are not included? Or prenatal/postpartum care? (perhaps there are none, but a statement or elucidation on pregnancy testing as the sole domain might prevent confusion)

Results: I appreciate the importance of this method to capture complexity. What was unclear to me as a reader is how Table 5: Ten ICOMOCs, divided into Planning/Doing/Sustaining then became the subheadings: Teaming; Assessing Needs and Capacity; etc. etc.?

Suggest mapping out ICOMOCs with the sub-headings used, perhaps an additional table is warranted for this

What do the authors mean by line 30-31 p. 15 "should include prison staff (A3) as the gatekeepers to access”? This is concerning as it is unclear whether they mean clinical/healthcare prison staff? This suggests the studies reviewed identify non-clinical prison staff as gatekeepers to access as a positive practice

Lines 1-13, and 47-55 on p.17 offer clearly outlined finding, very strong

I am unfamiliar with this methodology so perhaps it is not possible, but I wonder whether the term “penetration” (O5) is absolutely necessary here, or could be adapted given the vulnerability of the population and sensitive nature of sexual and reproductive health

Language consistency: are "prison regime" and "prison estate" used interchangeably? What exactly is meant by each?

Refined program theory: could be elaborated on and a link to the table provided/embedded.

Discussion: suggest adding a table of the three overarching themes. These three themes are strong and important, would like to see them emphasized

Line 18 p. 19 who is meant by “deliverers”? Specifically health deliverers? Suggest consistency throughout

Same with “Programme planners” who exactly are these?

Surprised to read “Deliverers can feel unsafe” in summary of findings as this is the first instance that was identified. Invite authors to push back on myths that prison is a location for social support (as mentioned: lines 21-24 p. 4); as well as that people in prison are dangerous and pose a threat to healthcare providers.

Please use person-first language throughout (p. 20 line 7 "drug users")

Recommendations: a) who is "workforce" in this context? Prison? Healthcare? Researchers?

Diagrams p. 37-28:

P. 37 suggest putting headings above vs. below and in bold

Both pages need titles as well as to be linked/embedded within article

---

**Reviewer**	**2**
**Name**	**Grubb, Laura**
**Affiliation**	**Boston Children's Hospital, Adolescent Medicine**
**Date**	**27-Jan-2026**
**COI**	**None**

Appreciate the research into this underrepresented population. There are a few major elements that I suggest the authors address to make this paper more interesting and relevant for publication.

1. Strengthen the writing style by engaging the Active Verb tense as much as possible instead of the Passive Verb tense (https://owl.purdue.edu/owl/general_writing/academic_writing/active_and_passive_voice/active_versus_passive_voice.html)

2. In the Rationale section, recommend authors provide clear presentation of the evidence and avoid editorializing.

3. Simply explanations and streamline concepts. Throughout the manuscript, this reviewer felt about lost in the weeds while reading it.

Some additional items:

Please provide the following clarification:

What is "grey literature"?"

What does the term women include in the search? Cisgender identifying women only or others that also identify as women?

What are "snowballing methods?"

On page 4, lines 9-10 consider using the terms commercial and sex trafficking.

Page 4, please provide a clear distinction between jails and prisons and clarify if you searched/included both sites.

The graphics, conclusions, and discussion are quite strong.

**Reviewer**	**3**
**Name**	**Caswell, Rachel**
**Affiliation**	**University Hospitals Birmingham NHS Foundation Trust, Rachel Caswell**
**Date**	**04-Mar-2026**
**COI**	**None**

Dear Authors,

I thought using a realist review was useful and appropriate for the research question. Its use added to the findings and conclusions of this paper. I thought you explained the steps in the review well and that it followed RAMESES guidance.

I have a few minor queries and suggestions. Please see attached file.

Best wishes, Rachel Caswell

**** **The reviewer provided a marked copy with additional comments. Please contact the publisher for full details.** ****

VERSION 1 - AUTHOR RESPONSE

Editor / Reviewer	Location in Manuscript	Editor / Reviewer Comment	Response / Clarification by Lead Author
Reviewer 1	All	Suggest mentioning of gender diversity early on and use of inclusive language throughout.	The term 'women in prison' has been replaced by 'people in prisons that detain women' or PPDWs (and likewise for men). This is intended to be inclusive of gender diverse individuals in both male and female settings, adopting person-first language. Where possible, I define interventions as 'women's sexual and reproductive health', rather than by participant gender, to be inclusive and to acknowledge policy variations in how eligibility is defined for gender diverse people. 'Other women' has been replaced by 'those in the community'.<br>Further clarification is added to the description of the search strategy, and the lack of searches for non-binary and trans people has been added to discussion of the limitations.
Reviewer 1	p.4 lines 21-24	The claim that prison removes people from harmful environments is somewhat problematic.	This implication was not intended - the intent was to acknowledge that time in prison may present a 'less tumultuous' time for some, and therefore an opportunity for engagement with health and healthcare (Emerson et al. 2022). I have reinstated a sentence from an earlier draft to address this - this emphasises time in prison as an opportunity for engagement, and the principle of equivalence (see below).
Reviewer 1	p.4 lines 21-41	The potential for prisons to offer healthcare (which they are mandated to provide) could be made more clear to be an as-of-yet-unrealized opportunity.	This is addressed by reinstating a sentence from an earlier draft which introduces equivalence as a key principle (see above). The remainder of the paragraph eludes to the lack of evidence that this opportunity has been effectively realised, so no further revisions have been made.

Editor / Reviewer	Location in Manuscript	Editor / Reviewer Comment	Response / Clarification by Lead Author
Reviewer 1	pp.3-4	Rationale: clear and concise definition of realist synthesis would be suitable at the beginning of this section.	RAMESES publication guidelines for realist syntheses (Wong et al., 2013) recommend that the introduction section includes the rationale for conducting a review and the objectives, and that the methods section includes the rationale for realist synthesis. This was modified to bring the rationale for realist synthesis into the introduction section (between the rationale and the objectives) to help the reader understand the complexity of prisons as a context for implementation before setting out why this makes realist synthesis the most appropriate method to use. Having tested different ways of ordering the text, we felt this had the best flow. Therefore, no change has been made.
Reviewer 1	p.5 line 56 to p.6 line 10	Materials and methods: suggest including detail on author and research team involvement here.	Author roles added to the methods overview and throughout the materials and methods section. Further detail is already included in the 'contributor' section of the manuscript.
Reviewer 1	p.6 lines 1-2	“Scoping of the literature" was this done formally/informally? What exactly did this entail?	This scoping was informal and exploratory, as defined in item 7 of the RAMESES publication standards for realist syntheses (Wong et al., 2013). It involved reading key national and international policy/guidance documents relevant to women’s sexual and reproductive health interventions in prisons, and exploratory searches of online academic databases and google scholar to test and refine the search strategy. I have adjusted the wording in the manuscript to reflect this better.
Reviewer 1	p.5 line 56 to p.6 line 10	Suggest refining the introductory paragraph of methods section to include sub-headings and explanation of the sequence in which methods were conducted.	In the introductory paragraph, the terms 'initial…', 'next…', and 'finally…' are used to imply the sequence. Thereafter, the sub-headings used throughout the methods section align with the RAMESES publication standards, and are broadly sequential - given the iterative nature of developing and conducting a realist synthesis, being more specific about the sequence is problematic. Therefore, no changes have been made.
Reviewer 1	p.7 lines 17-18	Search strategy: how were the databases selected?	Databases were selected to provide an appropriate spread of the indexed health research literature, including medical, public health and mental health research.
Reviewer 1	p.7 lines 41-44	Who was the “other reviewer” that conducted the 10% secondary screening?	This is included in the 'contributor' section, later in the paper - a link to this section has been added to the selection and appraisal of documents section.
Reviewer 1	p.8 lines 18-29	Who conducted data extraction? Analysis? Discussion “in relation to theories and IPT to derive insights".	This has been clarified in the materials and methods overview section (see above), adopting the active verb tense.

Editor / Reviewer	Location in Manuscript	Editor / Reviewer Comment	Response / Clarification by Lead Author
Reviewer 1	p.6 lines 18-34	Regarding women’s sexual and reproductive health domains, I am curious as to why interventions to improve access to abortion are not included? Or prenatal/postpartum care? (perhaps there are none, but a statement or elucidation on pregnancy testing as the sole domain might prevent confusion).	The core specification for women's health hubs in England (2024) was used as a starting point for the women's sexual and reproductive health domains - abortion is mentioned as a consideration, but not a core service. When cross-referenced with the prison policy and guidance documents, abortion was absent from two (WHO, 2021; NHS England, 2023) and specified as out of scope for one (BASHH, 2023). In the PRI (2021), it is mentioned only in the context of having occurred prior to detention, and as a potential reason for detention. In the NICE guidance (2016), it is only mentioned in the context of risk assessment conversations. It was therefore not included.<br>Although preconception is specified as a core service for community-based women's health hubs (2024), it was absent from all five prison-specific documents, and so was removed.<br>Reflection that this method of selecting health domains may have been a limitation of the search strategy has been added to the strengths and limitations section.
Reviewer 1	pp.13-14, table 5	I appreciate the importance of this method to capture complexity. What was unclear to me as a reader is how Table 5: Ten ICOMOCs, divided into Planning/Doing/Sustaining then became the subheadings: Teaming; Assessing Needs and Capacity; etc. etc.?	The sub-headings were guided by the constructs specified within the CFIR implementation process domain - these are already included in the IPT graphic in supplementary file 1. The constructs were grouped into planning, doing, and sustaining to aid conceptualisation of implementation phases. In the interests of remaining succinct, no changes have been made to the methods - IPT section, however changes made to table 5 will hopefully make this more clear.
Reviewer 1	pp.13-14, table 5	Suggest mapping out ICOMOCs with the sub-headings used, perhaps an additional table is warranted for this.	Table 5 has been amended to include the sub-headings.
Reviewer 1	p.15 lines 30-31	What do the authors mean by "should include prison staff (A3) as the gatekeepers to access”? This is concerning as it is unclear whether they mean clinical/healthcare prison staff? This suggests the studies reviewed identify non-clinical prison staff as gatekeepers to access as a positive practice.	This implication is not intended. The studies showed that non-clinical prison staff often play an important role in facilitating access to interventions, especially when escorts are required. Often clinical staff cannot go to residential wings or areas of work, and so prison staff are literal gatekeepers. Sharing information with prison staff is often overlooked (if the focus is only within healthcare departments), but when this does happen, it helps them to perceive the benefits of the intervention, and they may be more motivated to facilitate access to the intervention for people in prison (within the context of prioritising security, regime, etc.).
Reviewer 1	pp.13-14, table 5	I am unfamiliar with this methodology so perhaps it is not possible, but I wonder whether the term “penetration” (O5) is absolutely necessary here, or could be adapted given the vulnerability of the population and sensitive nature of sexual and reproductive health.	The term penetration is specified in Proctor et al.'s implementation outcomes (2011) - no clear alternative term is suggested, other than perhaps spread or institutionalisation, which are too narrow in definition and not without negative connotations themselves. Replacing penetration with 'integration and reach' as the two main components seems the most appropriate and has been applied throughout.

Editor / Reviewer	Location in Manuscript	Editor / Reviewer Comment	Response / Clarification by Lead Author
Reviewer 1	p.15 line 8, and p.20 lines 24 and 53	Language consistency: are "prison regime" and "prison estate" used interchangeably? What exactly is meant by each?	Prison regime refers to the daily routine of a prison, including the schedule of activities (education, work, meals, cell time, etc.).<br>Prison estate refers to the physical sites, facilities and administration of the network of prisons.<br>Brief clarifications have been added.
Reviewer 1	p.18 line 56 to p.19 line 5	Refined program theory: could be elaborated on and a link to the table provided/embedded.	The refined programme theory is elaborated on in figure 3, which would be embedded should the manuscript be published. This was submitted as an additional file, as per BMJ Open requirements.
Reviewer 1	p.19	Discussion: suggest adding a table of the three overarching themes. These three themes are strong and important, would like to see them emphasized.	BMJ Open specify a maximum of 8 figures and tables. The manuscript already includes 5 tables and 3 figures, so adding an additional table is not permissible.
Reviewer 1	p.19 line 18, and p.19 line 29	Who is meant by “deliverers”? Specifically health deliverers? Suggest consistency throughout. Same with “Programme planners” who exactly are these?	The actor type 'deliverer' refers to any individual who directly delivers the intervention to the participant. This is often a healthcare worker, but could also include researchers and peers. Deliverers may work within the prison, visit the prison only for the purposes of delivering the intervention, or work in a community setting that provides 'outside' appointments to people in prison. A clarification has been added to first use of the term.<br>Programme planners was intended to refer to any actor type involved in planning implementation, and so the wording has been updated to reflect this. Again, specifics are not given as this was carried out by a range of different people in different roles, depending on the intervention and study type.
Reviewer 1	p.19 line 18	Surprised to read “Deliverers can feel unsafe” in summary of findings as this is the first instance that was identified. Invite authors to push back on myths that prison is a location for social support (as mentioned: lines 21-24 p. 4); as well as that people in prison are dangerous and pose a threat to healthcare providers.	ICAMOC3 assessing needs and capacity through formative research includes the sentence 'workforce capacity influenced implementation, encompassing skills/specialisms, training needs, time, engagement, and concerns such as fearing needlestick injury' - the latter was observed in Crowley et al. (2019). The implication that deliverers feel unsafe as a norm was not intentional, so the word has been changed to uncertain. On reflection, this better reflects the range of concerns or unknowns that people working in prisons may have to contend with.
Reviewer 1	p.20 lines 6-7	Please use person-first language throughout (p. 20 line 7 "drug users").	Drug users has been changed to people who use drugs.
Reviewer 1	p.20 line 45	Recommendations: a) who is "workforce" in this context? Prison? Healthcare? Researchers?	Clarified to be delivery workforce. Again, the lack of specificity is to acknowledge the breadth of people who may be involved in delivering women's sexual and reproductive health interventions in prisons.
Reviewer 1	p.37	Diagrams - suggest putting headings above vs. below and in bold.	Noted and amended accordingly.
Reviewer 1	pp.37-38	Both pages need titles as well as to be linked/embedded within article.	Noted and amended accordingly.
Reviewer 2	N/A	Strengthen the writing style by engaging the Active Verb tense as much as possible instead of the Passive Verb tense (https://owl.purdue.edu/owl/general_writing/academic_writing/active_and_passive_voice/active_versus_passive_voice.html).	This has been actioned throughout the manuscript, primarily in the introduction, and materials and methods.
Reviewer 2	pp.3-4	In the Rationale section, recommend authors provide clear presentation of the evidence and avoid editorializing.	Several sentences have been amended to remove language that may be deemed as editorialising.

Editor / Reviewer	Location in Manuscript	Editor / Reviewer Comment	Response / Clarification by Lead Author
Reviewer 2	N/A	Simply [simplify] explanations and streamline concepts. Throughout the manuscript, this reviewer felt about lost in the weeds while reading it.	It is hoped that the changes made to the structure and language of the manuscript address this concern.
Reviewer 2	p.7 line 14	What is "grey literature"?	To avoid confusion, we have changed this to 'unpublished' literature throughout.
Reviewer 2	p.7 lines 11-25	What does the term women include in the search? Cisgender identifying women only or others that also identify as women?	The search used the terms 'women', 'woman', and 'female'. The categorisation of prisons in England uses the binary male/female, and policy on the assignment of gender diverse individuals to these settings has changed over the time period in scope (and differs between the countries included in the search). Therefore, this binary terminology was deemed appropriate for searches that would include all participants within settings that detain women, whilst not excluding interventions which may have been implemented for individuals within a male prison who identify as female. Non-binary and transgender terms were not specified in the search, and this reflection has been added to the study limitations (however we found no studies in exploratory scoping or searches that targeted these group specifically at the exclusion of 'women' or 'females').
Reviewer 2	p.7 line 16	What are "snowballing methods"?	To avoid confusion, I have changed this to 'citation searching' throughout.
Reviewer 2	p.4 lines 9-10	Consider using the terms commercial and sex trafficking.	Added.
Reviewer 2	p.4 line 60	Please provide a clear distinction between jails and prisons and clarify if you searched/included both sites.	The searching process is detailed in the methods section - I have amended this to clarify that multiple search terms were identified through the informal scoping. The reader is signposted to view supplementary file 1 for full details of the search terms used.<br>A large number of synonyms for places of detention were found (including jail and prison), with variance in terms and usage between countries. As this paper is written for the context of England, the term prison is used throughout the manuscript, which aligns with national policy.
Reviewer 3	p.6 lines 1-2	This scoping exercise referenced here does not seem particularly related to the current realist review. How was it used to inform the IPT?	Revised to clarify that the exploratory searches identified key policy and guidance documents that included best practice for implementing women's sexual and reproductive health interventions in prisons, which were used to inform the IPT.
Reviewer 3	p.8 line 20	I tend to agree with your earlier statement on how you analysed the data from your literature searches, 'data were extracted into ICAMO configurations' rather than using a 'grounded theory approach'. You have an ITP [IPT], and are considering from the literature how and why specific contexts trigger mechanisms. However, I am aware you are in part generating theory at this stage in the	The IPT was informed by policy and guidance documents. Using a grounded theory approach was intended to identify contextual factors from real world studies that may have been missing from the IPT, with the potential to generate and/or refine theory. The sentence has been amended to help clarify this.

		review and grounded theory has been used in this way before.	
Reviewer 3	p.8 lines 31-35	No stakeholder or advisory group involvement?	This was unfortunately not possible within the time and resources available. This has been added as a limitation of the review.
Reviewer 3	p.20 line 23	I think the sentence would make more sense if you remove this comma? Please check.	Comma removed.

VERSION 2 - REVIEW

Reviewer 2

Name Grubb, Laura

Affiliation Boston Children's Hospital, Adolescent Medicine

Date 12-May-2026

COI

Again, recommend authors avoid editorializing in the Introduction/Background/Rationale. Present the Background and then let your study results lead to Conclusions/Discussion.

The authors describe prisons early in the publication and in the abstract. Does that mean that the literature search and additional research only looked at prisons and not jails? Prisons are typically the location of individuals who have been through the adjudication process. Jails house those who are detained but not fully processed and sentenced.

Again, recommend authors strengthen the writing style by engaging the Active Verb tense as much as possible instead of the Passive Verb tense

(https://owl.purdue.edu/owl/general_writing/academic_writing/active_a

nd_passive_voice/active_versus_passive_voice.html).

The authors wrote these sections particularly well: Strengths, Limitations, Discussion, and Conclusion. In the previous sections, I felt overwhelmed by the abbreviations and acronyms and struggled to stay engaged while reviewing.

Reviewer 3

Name Caswell, Rachel

Affiliation **University Hospitals Birmingham NHS Foundation Trust,**
Rachel Caswell

Date **28-Apr-2026**

COI

Thank you for resubmitting with revisions. You have answered and addressed my queries.

VERSION 2 - AUTHOR RESPONSE

Reviewer 2	N/A	Again, recommend authors avoid editorializing in the Introduction/Background/Rationale. Present the Background and then let your study results lead to Conclusions/Discussion.	Further amendments have been made to the rationale section of the introduction to avoid editorialising.
Reviewer 2	N/A	The authors describe prisons early in the publication and in the abstract. Does that mean that the literature search and additional research only looked at prisons and not jails? Prisons are typically the location of individuals who have been through the adjudication process. Jails house those who are detained but not fully processed and sentenced.	As all high-income countries were in scope, the search terms were not determined by any one country's judicial system. Instead, multiple search terms were used to include a broad range of potential places of detention, or descriptors of people in detention. I have amended the methods section to make this more explicit. The reader is also

				signposted to view supplementary file 1 for full details of the search terms used, but for clarity they were:<br>**Population:**<br>**Location:**<br>1. Subject heading search of 'prison'<br>2. (prison* OR jail* OR gaol* OR penitentia* OR reformatory OR watch?house* OR remand).ti,ab.<br>3. ((correction* OR justice OR judicial OR secure OR detention OR custodial OR penal) adj2 (system* OR centre OR center OR facilit* OR setting OR estate OR sentence)).ti,ab.<br>4. (inmate* OR convict* OR offender* OR criminal* OR felon* OR remand* OR detaine* Or (incarcerat* NOT hernia*)).ti,ab.<br>5. 1 Or 2 OR 3 OR 4<br>(NB: initial searches that

			included the term 'incarcerated' returned multiple studies about incarcerated hernias, hence 'not hernia' was specified under search line 4).
Reviewer 2	N/A	Again, recommend authors strengthen the writing style by engaging the Active Verb tense as much as possible instead of the Passive Verb tense (https://owl.purdue.edu/owl/general_writing/academic_writing/active_and_passive_voice/active_versus_passive_voice.html).	Further amendments have been made, primarily in the materials and methods section.
Reviewer 2	N/A	The authors wrote these sections particularly well: Strengths, Limitations, Discussion, and Conclusion. In the previous sections, I felt overwhelmed by the abbreviations and acronyms and struggled to stay engaged while reviewing.	Thank you. Where appropriate, I have replaced the following acronyms with the full terms: high-income countries (HIC), initial programme theory (IPT), programme theory (PT) and people in prisons that detain women (PPDW). However, in some places (particularly the main findings),

			replacing acronyms would risk making the text harder to follow rather than easier.<br>I have also made the ICAMOC headings within the main findings section bold in the hope that this helps the reader to navigate the manuscript better.
Reviewer 3	N/A	Thank you for resubmitting with revisions. You have answered and addressed my queries.	Thank you.
